# Development of Neuroregenerative Gene Therapy to Reverse Glial Scar Tissue Back to Neuron-Enriched Tissue

**DOI:** 10.3389/fncel.2020.594170

**Published:** 2020-11-05

**Authors:** Lei Zhang, Zhuofan Lei, Ziyuan Guo, Zifei Pei, Yuchen Chen, Fengyu Zhang, Alice Cai, Gabriel Mok, Grace Lee, Vishal Swaminathan, Fan Wang, Yuting Bai, Gong Chen

**Affiliations:** ^1^Department of Biology, Huck Institute of Life Sciences, Pennsylvania State University, University Park, PA, United States; ^2^Guangdong-Hongkong-Macau Institute of CNS Regeneration, Jinan University, Guangzhou, China

**Keywords:** brain repair, brain injury, NeuroD1, *in vivo* reprogramming, neuron to astrocyte ratio, neuroinflammation, blood-brain-barrier, astrocyte-to-neuron conversion

## Abstract

Injuries in the central nervous system (CNS) often causes neuronal loss and glial scar formation. We have recently demonstrated NeuroD1-mediated direct conversion of reactive glial cells into functional neurons in adult mouse brains. Here, we further investigate whether such direct glia-to-neuron conversion technology can reverse glial scar back to neural tissue in a severe stab injury model of the mouse cortex. Using an adeno-associated virus (AAV)-based gene therapy approach, we ectopically expressed a single neural transcription factor NeuroD1 in reactive astrocytes in the injured areas. We discovered that the reactive astrocytes were efficiently converted into neurons both before and after glial scar formation, and the remaining astrocytes proliferated to repopulate themselves. The astrocyte-converted neurons were highly functional, capable of firing action potentials and establishing synaptic connections with other neurons. Unexpectedly, the expression of NeuroD1 in reactive astrocytes resulted in a significant reduction of toxic A1 astrocytes, together with a significant decrease of reactive microglia and neuroinflammation. Furthermore, accompanying the regeneration of new neurons and repopulation of new astrocytes, new blood vessels emerged and blood-brain-barrier (BBB) was restored. These results demonstrate an innovative neuroregenerative gene therapy that can directly reverse glial scar back to neural tissue, opening a new avenue for brain repair after injury.

## Highlights

- The glial scar can be reversed back to neural tissue through neuroregenerative gene therapy
- Astrocytes are not depleted after neuronal conversion
- Neuron to glia ratio after an injury can be rebalanced through *in vivo* cell conversion
- Conversion of reactive astrocytes into neurons reduces neuroinflammation
- Astrocyte-to-neuron conversion restores blood vessels and blood-brain-barrier

## Introduction

A delicate balance between neurons and glial cells is critical to maintaining normal brain functions. Injuries in the central nervous system (CNS) often results in neuronal loss and glial proliferation, leading to severe impairment of neuron-glia balance. Restoration of the neuron-glia balance is rather difficult because adult mammalian brains have largely lost the neuroregeneration capability (Yiu and He, 2006; Cregg et al., 2014; He and Jin, 2016; Sorrells et al., 2018). Reactive glial cells after injury may serve as a physical barrier to prevent the spreading of injury (Barker et al., 2018), but they can also secrete neuroinhibitory factors such as chondroitin sulfate proteoglycans (CSPGs) and lipocalin-2 (LCN2), as well as inflammatory cytokines such as TNFα and interleukin-1β (IL-1β; Silver and Miller, 2004; Koprivica et al., 2005; Ferreira et al., 2015). Neutralization of the neuroinhibitory factors can facilitate axonal regeneration (Bradbury et al., 2002; Sivasankaran et al., 2004). Injuries in the CNS may also induce neuroinflammation and breakdown of blood-brain-barrier (BBB), resulting in a toxic microenvironment for neuronal and glial survival (Silver and Miller, 2004). Reduction of neuroinflammation in injury sites can effectively reduce neuronal death (Fu et al., 2015; Witcher et al., 2015). However, neural protection alone is not sufficient for tissue repair if there are a large number of neurons lost and replaced by a large number of glial cells in the injury sites.

We have recently demonstrated that ectopic expression of NeuroD1 in reactive astrocytes can directly convert them into functional neurons inside the mouse brain (Guo et al., 2014; Chen et al., 2020; Wu et al., 2020). Such glia-to-neuron conversion can also be achieved with other neural transcription factors, such as Sox2 (Niu et al., 2013; Heinrich et al., 2014; Su et al., 2014), Ngn2 (Grande et al., 2013; Heinrich et al., 2014; Gascón et al., 2016), and Ascl1 (Torper et al., 2013, 2015; Liu et al., 2015; Rivetti di Val Cervo et al., 2017) or knockdown PTBP1 (Qian et al., 2020). However, it is currently unclear what impact will the *in vivo* glia-to-neuron conversion have on the glial scar formation. Will glial cells be depleted after conversion? Will injury become worse after conversion? Will neuron-glia balance be restored after conversion?

To answer these questions regarding *in vivo* glia-to-neuron conversion, we employed a severe stab injury model in the mouse motor cortex to investigate the broad impact of cell conversion on the microenvironment of injured brains. We found that astrocytes were not depleted after conversion, but rather repopulated due to intrinsic proliferation capability. The neuron-glia ratio was rescued by astrocyte-to-neuron (AtN) conversion, and the microenvironment of the injury sites was ameliorated after conversion. Specifically, A1 type reactive astrocytes in the injury sites were transformed into less reactive astrocytes within 3 days of NeuroD1 infection. Reactive microglia were also ameliorated and neuroinflammation was reduced following NeuroD1-mediated AtN conversion. Furthermore, BBB was restored and new synaptic connections were established after AtN conversion. Together, we demonstrate that NeuroD1-mediated cell conversion can rebalance the neuron-glia ratio in the injury sites and reverse glial scar back to neural tissue.

## Materials and Methods

### Mouse Model of Stab Injury and Virus Injection

Animal procedures were performed following the Animal Protection Guidelines of the US National Institutes of Health, and all experimental protocols were approved by the Pennsylvania State University’s Institutional Animal Care and Use Committee (IACUC). Wild type (WT) C57BL/6J and FVB/N-Tg(GFAP::GFP) 14Mes/J transgenic mice were purchased from Jackson Laboratory. Mice were housed in a 12 h light/dark cycle and supplied with sufficient food and water. Adult mice (20–30 g) with both genders were recruited in the experiments at the age of 3–6 months old. The mouse motor cortex was injured with a blunt needle (0.95 mm outer diameter) as previously reported with modifications (Bush et al., 1999; Bardehle et al., 2013; Guo et al., 2014). Briefly, ketamine/xylazine (100 mg/kg ketamine; 12 mg/kg xylazine) was administrated by intraperitoneal injection. Under anesthesia, mice were placed in a stereotaxic apparatus with the skull and bregma exposed by a midline incision. A hole of ~1.2 mm was drilled in the skull in both sides of the motor cortex (coordination: +1.0 mm anterior-posterior, ±1.5 mm medial-lateral relative to Bergma). A blunt needle (0.95 mm) was placed into each site to the depth of −1.8 mm dorsal-ventral and stayed still for 3 min. At 4 or 10 days post stab injury (dps) when astrocytes became reactive, mice were randomly subjected to either NeuroD1 or control virus-injection into the same site. The viral injection procedures were similar as previously reported (Guo et al., 2014), with each injection site received 1.5 μl adeno-associated virus (AAV) or retrovirus using a 5 μl micro-syringe and a 34 Gauge needle (Hamilton). The viral injection rate was controlled at 0.15 μl/min, with the needle gradually moved up at a speed of 0.1 mm/min. After injection, the needle was maintained in place for additional 3 min before fully withdrawn. Post-surgery, mice were recovered on a heating pad until free movement was observed. We did not observe any paralysis in mice after surgery. Mice were singly housed and carefully monitored daily for at least 1 week.

### Brain Section Inclusion and Quantification

To make sure the brain slides were comparable among different groups, we sectioned the brain in 40 μm thickness with vibrotome and store the slices in a 24-well plate with two slices per well in a rostral-to-caudal sequence. The slices with the maximal lesion were identified by a light microscope and marked as injury core. The brain slices with the same distance to the injury core among different groups were selected for comparison. Usually, only slices within ±0.2 mm distance away from the injury core were included for the current study.

To quantify cellular staining (NeuN, NeuroD1, GFAP, BrdU, LCN2, Iba1, iNOS, CSPG, and CD68), the entire lesioned cortical tissue was covered in 20× images (1.5 × 1.5 mm). The intensity or covered area was quantified in 20× images. To quantify the astrocyte and neuron numbers, a 0.3 × 1.5 mm region in the middle of the lesion area was selected. To quantify the neurites and synaptic puncta, high mag (40× or 63×) images were taken in the area at 0.3–0.5 mm away from the needle track, where the cell debris and nonspecific background were minimal.

ImageJ software was used for image quantifications. To quantify the intensity of GFAP, CSPG, Iba1, iNOS, and SMI32, the whole image was selected and the average intensity was quantified. Synaptic puncta were quantified by using the function of “analyze particles.”

### AAV Vector Construction

The hGFAP promoter was obtained from a pDRIVE-hGFAP plasmid (InvivoGen Inc.,) and inserted into pAAV-MCS (Cell Biolab) between MluI and SacII to replace the CMV promoter. The Cre gene was obtained by PCR from Addgene plasmid #40591 (gift of Dr. Albee Messing) and inserted into pAAV MCS between EcoRI and Sal1 sites to generate pAAV-hGFAP::Cre vector. To construct pAAV-FLEX-mCherry-P2A-mCherry and pAAV-FLEX-NeuroD1-P2A-mCherry vectors, the NeuroD1 or mCherry-coding cDNA was obtained by PCR using the retroviral constructs described previously (Guo et al., 2014). The NeuroD1 gene was fused with P2A-mCherry and subcloned into the pAAV-FLEX-GFP vector (Addgene plasmid #28304, a gift of Dr. Edward Boyden) between Kpn1 and Xho1 sites. Plasmid constructs were verified by sequencing.

### AAV Production

Recombinant AAV stereotype 9 was produced in 293AAV cells (Cell Biolabs). Briefly, triple plasmids [pAAV expression vector, pAAV9-RC (Cell Biolab) and pHelper (Cell Biolab)] were transfected by polyethyleneimine (PEI, linear, MW 25000). Cells were scraped and centrifuged at 72 h post-transfection. Cell pellets were frozen and thawed for four times by being placed in dry ice/ethanol and 37°C water bath alternately. AAV lysate was purified by ultra-centrifugation at 54,000 rpm for 1 h in discontinuous iodixanol gradients (Beckman SW55Ti Rotor). The virus-containing layer was collected followed by concentration by Millipore Amicon Ultra Centrifugal Filters. Virus titers were initially determined by QuickTiter^TM^ AAV Quantitation Kit (Cell Biolabs): 1.5 × 10^11^ – 1.2 × 10^12^ GC/ml for hGFAP::Cre, 1.4 × 10^12^ GC/ml for FLEX-NeuroD1-P2A-mCherry, 1.6 × 10^12^ GC/ml for FLEX-mCherry-P2A-mCherry.

### Retrovirus Production

The pCAG::GFP-IRES-GFP retroviral vector was a gift from Dr. Fred Gage (Salk Institute, CA, USA). Mouse NeuroD1 sequence was inserted into the above-mentioned vector to generate pCAG::NeuroD1-IRES-GFP vector (Guo et al., 2014). To package retroviral particles, the target vector with vesicular stomatitis virus glycoprotein (VSV-G) vector was transfected by PEI in gpg helper-free human embryonic kidney (HEK) cells. The titer of retroviral particles was determined as ~10^7^ particles/ml according to the serial dilution method.

### Tissue Collection

Brain samples were collected as previously described (Guo et al., 2014). Briefly, animals were injected with 2.5% Avertin for anesthesia. Transcardial perfusion with artificial cerebral spinal fluid (ACSF) was performed to systemically wash out the blood to better preserving the brain structure and antigen. After perfusion, brains were dissected out for immunohistochemistry or RT-PCR analyses. For immunohistochemistry, brains were post-fixed in 4% paraformaldehyde (PFA) at 4°C overnight, then washed with PBS and sectioned in 40 μm thickness. The brain slices were either used immediately or kept in PBS with 0.02% NaN_3_ in 4°C cold room for storage of about 1 month. For longer preservation, brain slices were transferred into anti-frozen solutions (50% xylene + 50% glycerol, at 1:1 mixture with MilliQ H_2_O) and kept at −20°C. For RNA extraction purposes, brain tissues around the injury site (~1.5 × 1.5 × 1 mm cube) were dissected out and flash-frozen at −80°C till usage.

### Immunohistochemistry

After fixation, brain tissues were sectioned at 40 μm sections using Leica-1000 vibratome. Brain slices were washed three times with phosphate-buffered saline (PBS) followed by permeabilization in 2% Triton X-100 in PBS for 1 h. Then brain sections were blocked in 5% normal donkey serum and 0.3% Triton X-100 in PBS for 1 h. The primary antibodies were added to the blocking buffer and incubated with brain sections overnight at 4°C. Primary antibodies were rinsed off with PBS three times followed by secondary antibody incubation for 2 h at room temperature (RT). After being washed with PBS, brain sections were mounted onto a glass slide with an anti-fading mounting solution containing DAPI (Invitrogen). Images were acquired with confocal microscopes (Olympus FV1000 or Zeiss LSM800). To ensure antibody specificity, the only secondary antibody was used for immunostaining as a side-by-side control, with no distinct signal detected. Primary antibodies used in the current study was listed in Table 1.

**Table 1:** Primary antibodies used in the study.

Primary antibody	Species	Company	Catalog#	Dilution
AQP4	Rabbit	Santa Cruz	Sc-20812	1:600
BrdU	Rat	Accurate Chemical	YSRTMCA 2060GA	1:600
CD68	Mouse	Abcam	Ab31630	1:600
Connexin43	Rabbit	Abcam	Ab11730	1:800
CSPG	Mouse	Sigma	C8035	1:600
GFP	Chicken	Abcam	Ab13970	1:1,000
GFAP	Rabbit	Millipore	AB5804	1:1,000
GFAP	Chicken	Millipore	AB5541	1:1,000
Iba1	Rabbit	Wako	019-19741	1:800
Iba1	Goat	Abcam	Ab5076	1:400
iNOS	Mouse	Millipore	MABN533	1:400
LCN2	Gt	R&D	AF1857	1:600
LY6C	Rat	Abcam	AB15627	1:800
MAP2	Chicken	Abcam	AB5392	1:400
MBP	Chicken	Millipore	AB9348	1:400
NeuN	Rabbit	Millipore	ABN78	1:1,000
NeuroD1	Mouse	Abcam	AB60704	1:600
NG2	Mouse	Millipore	MAB5384	1:400
RFP	Rat	Antibodies-online.com	ABIN334653	1:1,000
S100b	Mouse	Abcam	Ab66028	1:800
SMI32	Mouse	Biolegend	801701	1:1,000
SMI312	Mouse	Biolegend	SMI312R	1:1,000
SV2	Mouse	DSHB	SV2	1:1,000
vGlut1	Rabbit	Synaptic system		1:1,000
GAD67	Mouse	Millipore	MAB5406	1:1,000

### BrdU Labeling

For labeling of proliferative astrocytes after brain injury, GFAP-GFP transgenic mice were used and intraperitoneal injection of BrdU (Invitrogen) was conducted daily from 1 dps to 4 or 10 dps at a dose of 0.1 ml/10 g. For characterization of newly generated astrocytes after conversion, BrdU was administrated daily from 7 to 14 dpi. Fixed brain sections were subjected to 30 min treatment with 2 M HCl at 37°C for DNA denaturation. After five washes with PBS, brain sections were permeablized in 2% Triton-PBS for 1 h and incubated in blocking buffer (5% normal donkey serum and 0.3% Triton in PBS) for an additional 1 h at RT. Primary antibodies were mixed in a blocking solution and incubated with brain sections at 4°C overnight.

### Cresyl Violet Staining (Nissl Staining)

A Series of coronal sections were collected for analysis of tissue damage. The center of needle injury was collected and set as zero points; two sections anterior and posterior to the injury site at 200 μm intervals were also selected. The brain sections were first placed in xylene for 5 min, followed by a gradual hydration series with alcohol (95, 70, and 0% in water) for 3 min each. The samples were transferred to cresyl violate buffer (0.121 mg/ml cresyl violet acetate in NaAc buffer, pH 3.5) for 8 min at 60°C. Upon completion, the stain was rinsed and dehydrated in a series with alcohol (0, 70, 95, and 100%) for 30 s each. Finally, the samples were cleared in xylene for 1 h and mounted in DPX Mountant (Sigma–Aldrich). Images were acquired using an Olympus BX61 microscope.

### RNA Extraction and Quantitative Real-Time PCR

The RNA extraction was performed using the Macherey-Nagel NucleoSpin RNA kit (Macherey-N Macherey-Nagel, Bethlehem, PA, USA). RNA concentration and purity were measured by NanoDrop. For cDNA synthesis, 500 ng RNAs were mixed with Quanta Biosciences qScript cDNA supermix and incubated at 25°C for 5 min, 42°C for 30 min, 85°C for 5 min and held at 4°C. Upon completion, the cDNAs were diluted 5-fold with RNase/DNase-free water. The primers for real-time qPCR were designed using Applied Biosystems Primer Express software and synthesized in IDT. Primers used in the current study was listed in Table 2. For qRT-PCR, each reaction, 6.25 μl SYBR Green Supermix (Quanta Biosciences PerfeCTa, ROX), 2 μl cDNA, and 3.75 μl water were mixed well and loaded in a 96-well plate (Applied Biosystem Inc.). *Gapdh* was used as an internal control. All comparisons were conducted to non-injured cortical tissue from healthy mice. The comparative Ct method was used for the calculation of fold change.

**Table 2:** Primers used in the study.

Primer	Forward sequence	Reverse sequence
m-Gapdh	GGAGCGAGACCCCACTAACA	ACATACTCAGCACCGGCCTC
m-NeuroD1	AAAGCCCCCTAACTGACTGCA	TCAAACTCGGCGGATGGTT
m-Gfap	TTCAGCCACACCTTTCCAGC	CCTTAGAGGAGGCCTGGGAG
m-Lcn2	AACTTGATCCCTGCCCCATCT	TTTCTGGACCGCATTGCCT
m-Gbp2	GGGGTCACTGTCTGACCACT	GGGAAACCTGGGATGAGATT
m-Serping1	ACAGCCCCCTCTGAATTCTT	GGATGCTCTCCAAGTTGCTC
m-Anax2	CAGGACATTGCCTTCGCCTAT	TAGGCCCAAAATCACCGTCTC
m-Thbs1	CGTGAGCGATGAGAAGGACA	CGATCTGTGCTTGGTTGTGC
m-Gpc6	CGGCCAGACACTTTCATCAGA	TGGATTCATCGCTTGTGTCTTG
m-S100a10	CCTCTGGCTGTGGACAAAAT	CTGCTCACAAGAAGCAGTGG
m-Tm4sf1	GCCCAAGCATATTGTGGAGT	AGGGTAGGATGTGGCACAAG
m-Il1b	TTGAAGTTGACGGACCCCAA	TGTTGATGTGCTGCTGCGA
m-Il6	TTCCATCCAGTTGCCTTCTTG	CATTTCCACGATTTCCCAGAG
m-Tnf	CACAAACCACCAAGTGGAGGA	ACAAGGTACAACCCATCGGCT

### Electrophysiology

Brain slice recordings were performed as previously described (Guo et al., 2014). Briefly, 1 month after NeuroD1-AAV injection, mice were anesthetized with 2.5% avertin, and then perfused with NMDG-based cutting solution (in mM): 93 NMDG, 93 HCl, 2.5 KCl, 1.25 NaH_2_PO_4_, 30 NaHCO_3_, 20 HEPES, 15 glucose, 12 *N*-Acetyl-L-cysteine, 5 sodium ascorbate, 2 Thiourea, 3 sodium pyruvate, 7 MgSO_4_, 0.5 CaCl_2_, pH 7.3–7.4, 300 mOsm, bubbled with 95% O_2_/5% CO_2_. Coronal sections of 300 μm thickness were cut around AAV-injected cortical areas with a vibratome (VT1200S, Leica, Germany) at RT. Slices were collected and incubated at 33.0 ± 1.0°C in oxygenated NMDG cutting solution for 10–15 min. Then, slices were transferred to holding solutions with continuous 95% O_2_/5% CO_2_ bubbling (in mM): 92 NaCl, 2.5 KCl, 1.25 NaH_2_PO_4_, 30 NaHCO_3_, 20 HEPES, 15 glucose, 12 *N*-Acetyl-L-cysteine, 5 sodium ascorbate, 2 Thiourea, 3 sodium pyruvate, 2 MgSO_4_, 2 CaCl_2_. Brain sections were recovered in the holding solution at least for 0.5 h at room temperature. For patch-clamp recording, a single slice was transferred to the recording chamber continuously perfused with standard ACSF (in mM: 124 NaCl, 2.5 KCl, 1.25 NaH_2_PO_4_, 26, NaHCO_3_, 10 Glucose, 1.3 MgSO_4_, 2.5 CaCl_2_) saturated by 95% O_2_/5% CO_2_ at 33.0 ± 1.0°C. To record action potentials and ionic currents, whole-cell recordings were performed with pipette solution containing (in mM): 135 K-Gluconate, 10 KCl, 5 Na-phosphocreatine, 10 HEPES, 2 EGTA, 4 MgATP and 0.3 Na_2_GTP, pH 7.3 adjusted with KOH, 280–290 mOsm. Depolarizing currents were injected to elicit action potentials under the current-clamp model. To record spontaneous excitatory postsynaptic currents (sEPSCs) and spontaneous inhibitory postsynaptic currents (sIPSCs), pipette solution contained (in mM): 120 Cs-Methanesulfonate, 10 KCl, 10 Na-phosphocreatine, 10 HEPES, 5 QX-314, 1 EGTA, 4 MgATP and 0.3 Na_2_GTP, pH 7.3 adjusted with KOH, 280–290 mOsm. The cell membrane potential was held at −70 mV for sEPSC recordings, and 0 mV for sIPSC recordings, respectively. Data were collected with a MultiClamp 700A amplifier and analyzed with pCLAMP10 software (Molecular Devices).

### Data Analysis and Statistics

Prism 6 Graphpad software was used for statistical analysis and bar graphs. ImageJ version 1.46r, Java 1.6.0_20 (64-bit) was used for imaging analysis. For comparison of two data sets, Paired Student’s *t*-test, two-tailed was conducted. For comparison of three data sets, one-way or two-way analysis of variance (ANOVA) was performed, followed by *post hoc* tests. Statistical significance was set as *p* < 0.05. Data were presented as mean ± SEM.

## Results

### NeuroD1-Mediated Astrocyte-to-Neuron Conversion in a Severe Stab Injury Model

The functional role of reactive glial cells after a neural injury is still controversial despite extensive research over the past decades. On one hand, reactive glial cells have long been reported to secret neuroinhibitory and neuroinflammatory factors to hamper neuroregeneration (Bush et al., 1999; Silver and Miller, 2004; Yiu and He, 2006; Cregg et al., 2014; He and Jin, 2016); on the other hand, a glial scar may have a beneficial effect on axonal regeneration (Anderson et al., 2016; Silver, 2016). We have recently demonstrated that reactive astrocytes and NG2 cells can be directly converted into functional neurons inside mouse brains by a single transcription factor NeuroD1 (Guo et al., 2014). Since glial scar seems to be a double-edged sword, we wondered whether converting astrocytes into neurons might cause any detrimental effects and make the injury worse. To answer this question, we established a severe stab injury model in adult mice (3–6 months old, both genders included) and investigated the impact of NeuroD1-mediated astrocyte-to-neuron (AtN) conversion on the microenvironment of the injury areas. Specifically, we used a blunt needle (outer diameter 0.95 mm) to make a severe stab injury in the mouse motor cortex, which induced a significant tissue loss together with reactive astrogliosis in the injury sites (Supplementary Figure 1A). As expected, the number of astrocytes increased significantly in the stab-injured areas at 4 and 10 dps (Supplementary Figure 1A, right bar graph). This is consistent with the increased proliferation of astrocytes following neural injury, as shown by the incorporation of bromodeoxyuridine (BrdU) during cell division (Supplementary Figure 1B; BrdU^+^ astrocytes, 38.7 ± 2.5%, *n* = 4 mice, 10 dps). Also, the astrocytes accumulated in the lesion site showed hypertrophic elongated morphology and lost their own territory with processes overlapping with each other (Supplementary Figures 1A,B). Therefore, our severe stab injury model in the mouse motor cortex causes a significant tissue loss and formation of glial scar in the injury sites.

**Figure 1:**
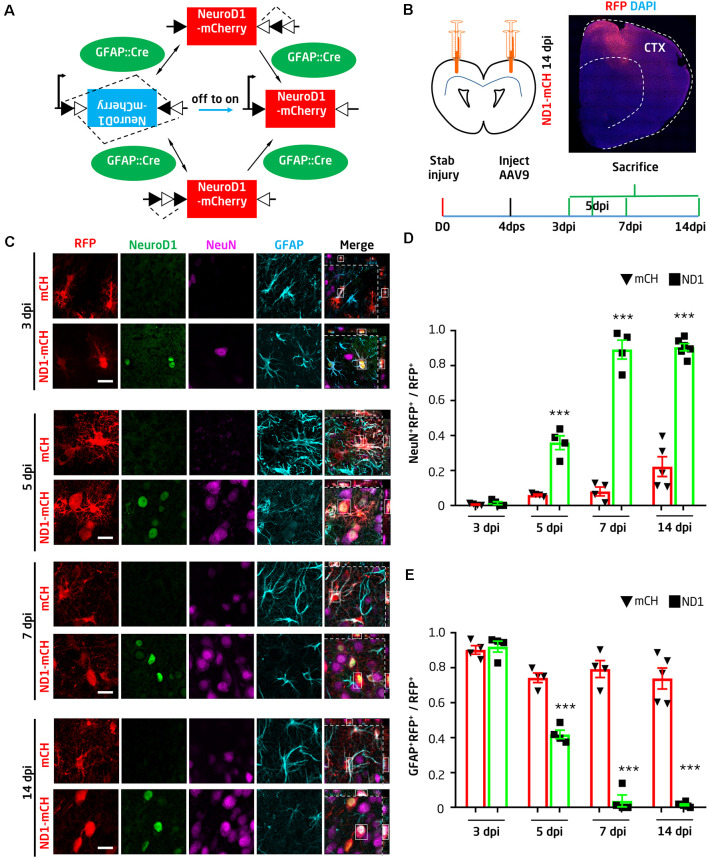
NeuroD1-mediated high-efficiency astrocyte-to-neuron conversion after stab injury. **(A)** Schematic illustration showing the Cre-FLEX system. GFAP::Cre viruses express Cre in astrocytes, where Cre acts at the loxP-type recombination sites of FLEX-CAG::NeuroD1-P2A-mCherry (inverted sequence). After Cre-mediated recombination, NeuroD1 will be expressed under the control of the CAG promoter. **(B)** Schematic illustration showing stab injury and adeno-associated virus (AAV) injection in both sides of the mouse motor cortex, followed by analyses at different time points. To evaluate the effect of NeuroD1-treatment, the AAV serotype 9 (AAV9) carrying GFAP::Cre and FLEX-CAG::NeuroD1-P2A-mCherry or FLEX-CAG::mCherry-P2A-mCherry (control) were injected into the injury site at 4 days post stab injury (dps). Mice were sacrificed at 3, 5, 7, or 14 days post-viral injection (dpi) for analyses. A half brain section with NeuroD1-AAV9 injection showed broad viral infection around the one side of the motor cortex area (top right). **(C)** Ectopic expression of NeuroD1 in reactive astrocytes after stab injury gradually shifted the astrocytic population into neurons. Representative images showing control AAV mCherry-infected cells with clear astrocytic morphology and GFAP signal (cyan) across different time points (3, 5, 7, and 14 dpi). In contrast, NeuroD1-infected cells (arrowheads) initially showed colocalization of NeuroD1 (green) and GFAP at 3 dpi, indicating infection of astrocytes, but later showed colocalization of NeuroD1 and NeuN (magenta) at 5, 7, and 14 dpi, suggesting conversion into neurons. Note that the astrocytic morphology in the NeuroD1 group also became less hypertrophic compared to the control group. Scale bar = 20 μm. **(D,E)** Quantitative analysis of the ratio of neurons vs. astrocytes among all virally infected cells in both control (black bar) and NeuroD1 group (gray bar). At 3 dpi, the majority of mCherry- and NeuroD1-infected cells remained as GFAP^+^ astrocytes; at 5 dpi, NeuN^+^ started to be observed in NeuroD1-infected cells; at 7 and 14 dpi, while control mCherry-infected cells remained GFAP^+^, the majority of NeuroD1-infected cells became NeuN^+^ neurons. Note that at 14 dpi, some control mCherry-infected cells were NeuN^+^, suggesting that some injured neurons might have GFAP-Cre and FLEX-mCherry expression. *n* = 4–6 mice per group. Each dot represents one animal. ***P < 0.001, two-way analysis of variance (ANOVA) followed by Bonferroni *post hoc* test.

To convert glial cells into neurons, we first employed retroviruses expressing NeuroD1 in dividing reactive glial cells as previously reported (Guo et al., 2014). We injected retroviruses into the injury site at 10 dps and collected the tissue at 14 days post-viral injection (dpi). We confirmed that ectopic expression of NeuroD1-GFP in glial cells efficiently (90.6 ± 5.2%) converted them into NeuN^+^ neurons, whereas none of the GFP-infected cells were co-labeled by NeuN in the control group (Supplementary Figure 2, *n* = 4 mice). Despite high conversion efficiency, the total number of newly converted neurons after the retroviral infection was limited due to the limited number of glial cells that happened to be dividing during retroviral injecting. To increase the total number of newly converted neurons in the injury sites for therapeutic repair, we developed an AAV Cre-FLEX system to achieve more broad viral infection and cell conversion because AAV can express target genes in both dividing and non-dividing cells (Ojala et al., 2015). Specifically, Cre recombinase was expressed under the control of astrocyte promoter GFAP (GFAP:: Cre) to target astrocytes; the expression of Cre will act at the loxP-type recombination sites flanking an inverted sequence of NeuroD1-P2A-mCherry under the CAG promoter in a separate AAV vector (FLEX-CAG::NeuroD1-P2A-mCherry; Figure 1A). Therefore, NeuroD1 expression can be targeted to reactive astrocytes where the GFAP promoter is highly active, and the subsequent Cre-mediated recombination will lead to high expression of NeuroD1 driven by a strong promoter CAG. The use of a CAG promoter instead of a GFAP promoter is to ensure the detection of converted neurons through mCherry expression because the GFAP promoter will be gradually silenced after neuronal conversion. We have initially tried GFAP::NeuroD1-P2A-mCherry (or GFP), but it is difficult to identify the converted neurons after several weeks because they may have lost mCherry (or GFP) after the silence of GFAP promoter in converted neurons (data not shown).

The AAV Cre-FLEX system was proved to be very efficient as shown by the wide expression of NeuroD1-mCherry in the stab-injured cortical areas (Figure 1B). Because AAV can infect both neurons and glial cells in the brain, we first investigated whether our GFAP::Cre/FLEX system can target astrocytes as we designed. At 4 dps, AAVs expressing either GFAP::Cre/FLEX-mCherry or GFAP::Cre/FLEX-NeuroD1-mCherry were injected at the injury sites and brain sections were immunostained with GFAP and NeuN to assess the AAV-infected cell identity (Figure 1C). At 3 days of post-viral infection (dpi), both the control AAV mCherry and the NeuroD1-mCherry-infected cells were astrocytes (Figure 1C, top two rows). At 5 dpi, however, while the majority of control AAV mCherry-infected cells remained GFAP^+^ astrocytes, some of the NeuroD1-mCherry-infected cells started to show NeuN^+^ signal (Figure 1C, middle rows of 5 dpi). At 7 and 14 dpi, the NeuroD1-mCherry-infected cells showed stronger NeuroD1 expression (green) and NeuN signal (magenta), while the majority of control mCherry-infected cells remained astrocytes (Figure 1C, bottom rows of 7 and 14 dpi). The quantitative analysis found that at 3 days post-NeuroD1-mCherry viral injection (3 dpi), 92.1 ± 3.1% of NeuroD1-mCherry infected cells were GFAP-positive, indicating preferential infection of astroglial population guided by GFAP::Cre viruses. Among these NeuroD1-mCherry infected cells, 87.4 ± 2.5% were already immunopositive for NeuroD1 (Supplementary Figures 3A,B), suggesting a rapid expression of NeuroD1 using our AAV Cre-FLEX system. Such early expression of NeuroD1 was further confirmed by real-time PCR analysis (Supplementary Figure 4A). At 5 dpi, about 35.9 ± 3.9% of NeuroD1-infected astrocytes were converted into NeuN-positive neurons, while only 6.0 ± 0.4% of the control AAV mCherry-infected cells showed NeuN signal. At 7 dpi (injected at 4 dps), we found that 89.2 ± 4.7% of NeuroD1-infected cells showed NeuN signal (7 dpi, *n* = 4 mice), whereas in the control group 80% of mCherry AAV-infected cells were still GFAP^+^ astrocytes (Figures 1D,E). The number of NeuroD1-converted neurons in the injury areas were quantified as 219.7 ± 19.3/mm^2^ at 14 dpi. Together, by developing an AAV Cre-FLEX system to highly express NeuroD1 in reactive astrocytes, we achieved high efficiency of AtN conversion in the stab-injured mouse cortex. It is worth to mention that while GFAP::Cre may be expressed in radial glia or neuro progenitor, the current study is limited to the adult mouse cortex, where neuroprogenitor has rarely been demonstrated in 3-month old mice. Therefore, our neuronal conversion is most likely occurring in adult astrocytes, rather than adult neuro-progenitors which are mainly found in the subventricular zone of the hippocampal subgranular zone.

### Astrocytes Not Depleted After Conversion

**Figure 2:**
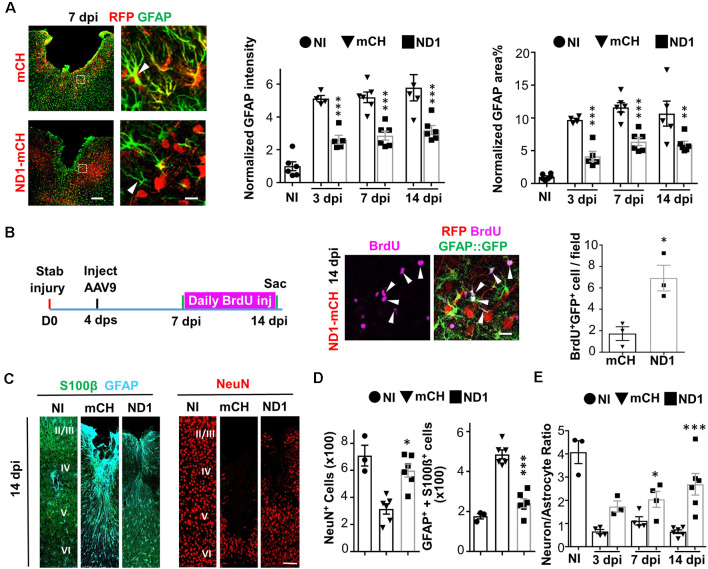
Rescue of neuron:astrocyte ratio through NeuroD1-mediated *in vivo* AtN conversion. **(A)** Astrocytes are not depleted in NeuroD1-converted areas. Control AAV-infected injury areas showed intensive GFAP signals (green) with hypertrophic morphology (top row, 7 dpi). In contrast, NeuroD1-infected injury areas showed significantly reduced GFAP expression, and astrocytic morphology was less reactive (bottom row, arrowhead). Astrocytes (green) persisted in the NeuroD1-converted area (red). Scale bar = 200 μm (low mag, left), and 20 μm (high mag, right). Quantitative analysis revealed a significant reduction of both GFAP intensity and GFAP-covered area in NeuroD1-infected injury areas (right bar graphs). Note that the GFAP signal in the NeuroD1 group was reduced to half of the control group but still higher than non-injured brains, indicating that astrocytes were not depleted after NeuroD1 conversion. *n* = 4–6 mice per group. Each dot represents one animal. **P < 0.01, ***P < 0.001, two-way ANOVA followed by Bonferroni *post hoc* test. **(B)** Increased proliferation of astrocytes after NeuroD1-mediated cell conversion. BrdU was applied daily in GFAP::GFP mice between 7–14 dpi, a time window of cell conversion, to assess cell proliferation. We detected many proliferating astrocytes that were co-labeled with BrdU (magenta) and GFP (green) in the vicinity of NeuroD1-converted neurons (red). Scale bar = 20 μm. Quantitative analysis revealed a significant increase in the number of proliferating astrocytes (BrdU^+^/GFP^+^) in NeuroD1-infected injury areas, compared to the control group. *n* = 3 mice. *P < 0.05, Student’s *t*-test. **(C)** Representative images illustrating astrocytes (S100β, green; GFAP, cyan) and neurons (NeuN, red) in non-injured brains (NI), and stab-injured brains with mCherry control virus infection (mCH) or NeuroD1 virus infection (ND1). Scale bar = 100 μm. **(D)** Bar graphs illustrating quantitative analyses of the number of NeuN^+^ neurons, and GFAP^+^/S100b^+^ astrocytes among non-injured, mCherry control, and NeuroD1 groups (14 dpi). **(E)** Rescue of neuron:astrocyte ratio after NeuroD1-mediated AtN conversion. Quantitation of neuron:astrocyte ratio among non-injured, mCherry control, and NeuroD1 groups at 3, 7, and 14 dpi. The neuron:astrocyte ratio was 4:1 in non-injured mouse motor cortex, but significantly decreased to 0.6:1 after stab injury, and then reversed back to 2.6:1 by NeuroD1-mediated AtN conversion. *n* = 3–6 mice per group. *P < 0.05, ***P < 0.001, two-way ANOVA plus *post hoc* Sidak’s test.

Astrocytes play a vital role in supporting neuronal functions (Allen and Barres, 2009). The high efficiency of AtN conversion raises a serious concern regarding whether astrocytes might be depleted (mostly absent) after neuronal conversion. We, therefore, performed GFAP staining and did observe an overall reduction of GFAP signal in the NeuroD1 group compared to the control group, but astrocytes were not absent in the converted area (Figure 2A). Furthermore, the astrocyte morphology showed a significant change in the NeuroD1 group, displaying much less hypertrophic processes compared to the control group (Figure 2A, arrowhead in left images), suggesting that astrocytes in the NeuroD1-converted areas became less reactive. The quantitative analysis found that compared to the non-injured cortex, the GFAP signal after stab injury was significantly increased by 5–10 folds in the mCherry control group (Figure 2A, bar graphs, black bar), but reduced significantly by about half in NeuroD1-infected areas (Figure 2A, gray bar). The decrease of the GFAP signal in the NeuroD1 group is consistent with the conversion of reactive astrocytes into neurons. On the other hand, our detection of a significant level of GFAP signal in the NeuroD1-infected areas suggests that reactive astrocytes are not depleted after conversion. Since astrocytes have an intrinsic capability to proliferate, we hypothesized that neuronal conversion might trigger the remaining astrocytes to proliferate to compensate for the loss of astrocytes undergoing conversion. To test this idea, we injected BrdU, which can be incorporated into DNA during cell division, daily from 7 dpi (viral injection at 4 dps) to 14 dpi to monitor cell proliferation in both the control group and NeuroD1 group (Figure 2B, left schematic illustration). We discovered that the number of BrdU-labeled astrocytes in the NeuroD1 group more than tripled that of the control group (Figure 2B, right panels, and quantified in the bar graph). Many of the BrdU-labeled astrocytes were adjacent to the NeuroD1-converted neurons (Figure 2B, arrowheads), suggesting that astrocytes can proliferate in the converted areas. Note that there were also astrocytes that were BrdU negative, suggesting that preexisting astrocytes and newly generated astrocytes intermingled together after AtN conversion. Therefore, astrocytes will not be depleted by AtN conversion but rather can be repopulated due to their intrinsic proliferative capability (Bardehle et al., 2013; Wanner et al., 2013).

### Neuron to Astrocyte Ratio Rebalanced After Conversion

Brain functions rely upon a delicate balance between neurons and glial cells. After the neural injury, both neurons and glial cells will die initially but only glial cells can proliferate following injury, resulting in an altered neuron: glia ratio in the injury areas. This was reflected in our severe stab injury model (Figure 2C), where the number of healthy neurons (NeuN^+^ cells, red) significantly decreased after injury (mCH column) but the number of astrocytes (GFAP^+^/S100b^+^) significantly increased in the lesion site (mCH column) throughout cortical layer II to VI (1.2 mm in depth and 0.3 mm in width). After NeuroD1-mediated AtN conversion, the NeuN^+^ neurons increased but the hypertrophic astrocytes decreased (Figure 2C, ND1 columns). Quantitatively, at 4 dps before viral injection, there were 316 ± 26 astrocytes (per 0.36 mm^2^ area) in the injury site. At 14 dpi, the number of astrocytes in the mCherry control group increased to 486 ± 22, but in the NeuroD1 group decreased to 241 ± 23, which was closer to the astrocyte number (176 ± 13) in the non-injured cortex. The fact that the number of astrocytes in the NeuroD1-converted areas was still more than that in non-injured areas further confirmed that the astrocytes were not depleted or absent after neuronal conversion. Such a decrease of astrocytes in the NeuroD1 group was accompanied by an increase of NeuN^+^ neurons (Figure 2D, the black square), as expected through AtN conversion. With simultaneous changes of both astrocytes and neurons after injury and AtN conversion, we further quantified the neuron: astrocyte ratio in the non-injured vs. injured cortex. We found that the neuron:astrocyte ratio in the mouse motor cortex was ~4:1 (four neurons to one astrocyte) in resting condition (Figure 2E, black dot, non-injury). After stab injury, the neuron:astrocyte ratio dropped to 0.6:1 (Figure 2E, black triangle, 3 dpi). After NeuroD1-conversion, the neuron:astrocyte ratio reverted to 2.6:1 at 14 dpi (Figure 2E, the black square). Given the critical role of a balanced neuron:astrocyte ratio in normal brain functions, a significant rescue of the neuron:astrocyte ratio after AtN conversion is an important step to restore normal brain functions.

### A1 Reactive Astrocytes Transformed During Conversion

**Figure 3:**
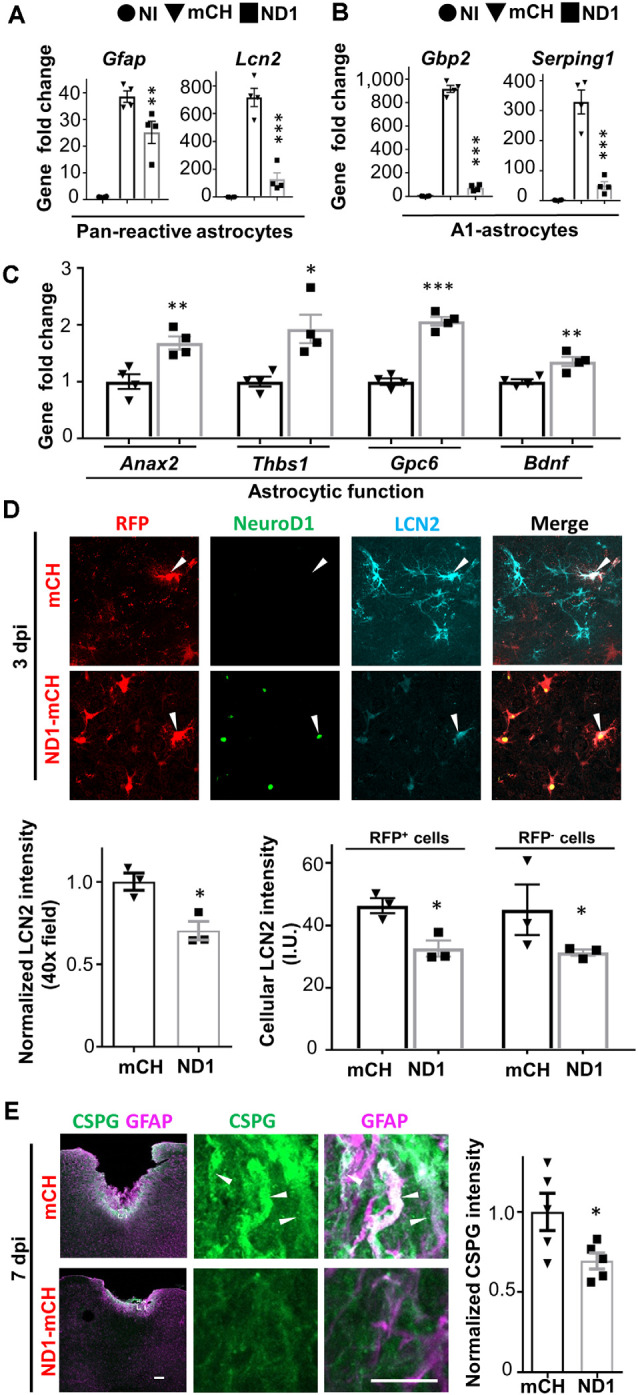
NeuroD1 transformed A1-type reactive astrocytes at an early point. **(A)** Quantitative real-time PCR (qRT-PCR) analysis revealed a dramatic increase of reactive astrocytic genes *Gfap* and *Lcn2* after stab injury, which was significantly attenuated in NeuroD1-infected areas. *n* = 4 mice. Each dot represents one animal. **P < 0.01, ***P < 0.001, one-way ANOVA followed with Sidak’s test. **(B)** A1 type astrocyte genes *Gbp2* and *Serping1* were upregulated several hundred folds in stab-injured cortices compared to non-injured cortices, but markedly reduced in NeuroD1-infected cortices. *n* = 4 mice. Each dot represents one animal. ***P < 0.001, one-way ANOVA followed with Sidak’s test. **(C)** More quantitative analyses with qRT-PCR revealed a significant upregulation of astrocytic functional genes in NeuroD1-infected cortices compared to the mCherry control group. *n* = 4 mice. Each dot represent one animal. *P < 0.05, **P < 0.01, ***P < 0.001, Student’s *t*-test. **(D)** Representative images showing that in control-AAV infected injury sites, neural injury marker lipocalin-2 (LCN2) was highly expressed (top row, arrowhead; 3 dpi). In contrast, the NeuroD1-infected area showed a much-reduced LCN2 signal (bottom row, arrowheads). Scale bar = 20 μm. Bar graphs: quantitative analyses revealed a reduced LCN2 expression in both NeuroD1-infected or non-infected cells compared to the control-AAV infected/non-infected cells. *n* = 3 mice. Each dot represents one animal. *P < 0.05, Student’s *t*-test. **(E)** Representative images showing the high expression level of chondroitin sulfate proteoglycan (CSPG) in reactive astrocytes in control-AAV infected injury sites (upper panels, arrowheads; 7 dpi), whereas NeuroD1-infected areas showed significantly reduced CSPG signal (lower panels). Scale bar = 200 μm (low mag, left), 10 μm (high mag, right). Bar graph showing quantitative analysis of the CSPG signal in control and NeuroD1 groups. *n* = 3 mice. Each dot represents one animal. *P < 0.05, Student’s *t*-test.

A recent study suggested that reactive astrocytes after injury or disease might be characterized by A1 and A2 astrocytes with different gene expression profiles (Liddelow et al., 2017). Specifically, A1 astrocytes were induced by inflammatory cytokines and exhibited neurotoxic effects, whereas A2 astrocytes were activated post-stroke and secret both neural inhibitory and neuroprotective factors. If astrocytes persisted after NeuroD1-mediated conversion, are they different from the reactive astrocytes in the control group? To answer this question, we collected brain tissue in lesion site (1.5 × 1.5 × 1 mm) and performed RT-PCR analysis of a variety of genes related to A1 astrocytes and neuroinflammation at 3 dpi, an early stage of neuronal conversion. Compared to the non-injury (NI) group, stab injury caused an upregulation of the pan-reactive astrocyte genes such as *Gfap* by 37-fold (Figure 3A), which was significantly attenuated in the NeuroD1 group (one-way ANOVA, Sidak’s test, **P < 0.01, *n* = 4 pairs). Lcn2, a neuroinflammation marker associated with reactive astrocytes after injury (Zamanian et al., 2012), was increased by 700-fold after stab injury, but drastically reduced in the NeuroD1 group (Figure 3A, one-way ANOVA, Sidak’s test, ***P < 0.001, *n* = 4 pairs). Also, genes characteristic for A1 astrocytes such as *Gbp2* and *Serping1* were upregulated by 300–900 folds after stab injury (one-way ANOVA, Sidak’s test, ***P < 0.001, *n* = 4 pairs), but greatly attenuated by an order of magnitude after NeuroD1 treatment (Figure 3B). Besides, NeuroD1 treatment also increased the astrocytic genes that support neuronal functions such as *Anax2, Thbs1, Gpc6, and Bdnf* (Figure 3C). As a validation, the expression of NeuroD1 in injured tissue was confirmed by RT-PCR analysis, showing a significant increase compared to the control group (Supplementary Figure 4A). Different from A1 astrocyte genes, the A2 reactive astrocyte genes were not changed despite a decrease of GFAP expression after NeuroD1-infection (Supplementary Figures 4B,C).

To determine how NeuroD1 expression affected the glial reactivity, we looked into the cellular changes by performing immunostaining at 3 dpi, at which time point NeuroD1 was already detected in infected astrocytes. Consistent with RT-PCR analysis in Figure 3A, we observed a global reduction in LCN2 intensity in the NeuroD1-infected injury areas (Figure 3D, Student’s *t*-test, *P < 0.05, *n* = 3 pairs). Next, we asked whether this LCN2 reduction is restricted only in NeuroD1 virus-infected cells. The quantitative analysis found that both NeuroD1-infected and non-infected astrocytes in the injured areas showed a significant decrease of LCN2 compared to the control group (Figure 3D, Student’s *t*-test, *P < 0.05, *n* = 3 pairs). Similarly, we observed a significant reduction of the GFAP level in both NeuroD1-infected and non-infected astrocytes (Supplementary Figure 4C, Student’s *t*-test, ***P < 0.001, ****P < 0.0001, *n* = 4 pairs). Therefore, NeuroD1-mediated reduction of astrocytic reactivity may be achieved through both cell-autonomous and non-cell-autonomous effects. Furthermore, CSPG is widely associated with reactive astrocytes after neural injury and plays an important role in neuron inhibition during glial scar formation (Koprivica et al., 2005). In our severe stab injury model, we detected a high level of CSPG in the injury areas (Figure 3E, top row); but in the NeuroD1-treated group, the CSPG level was significantly reduced (Figure 3E, bottom row; quantified in a bar graph, Student’s *t*-test, *P < 0.05, *n* = 5 pairs). Together, these results suggest that ectopic expression of NeuroD1 in reactive astrocytes significantly attenuated their reactive and neuroinflammatory properties, and such beneficial effects occurred as early as 3 days after NeuroD1 infection.

### Astrocyte-Microglia Interaction During *In vivo* Cell Conversion

**Figure 4:**
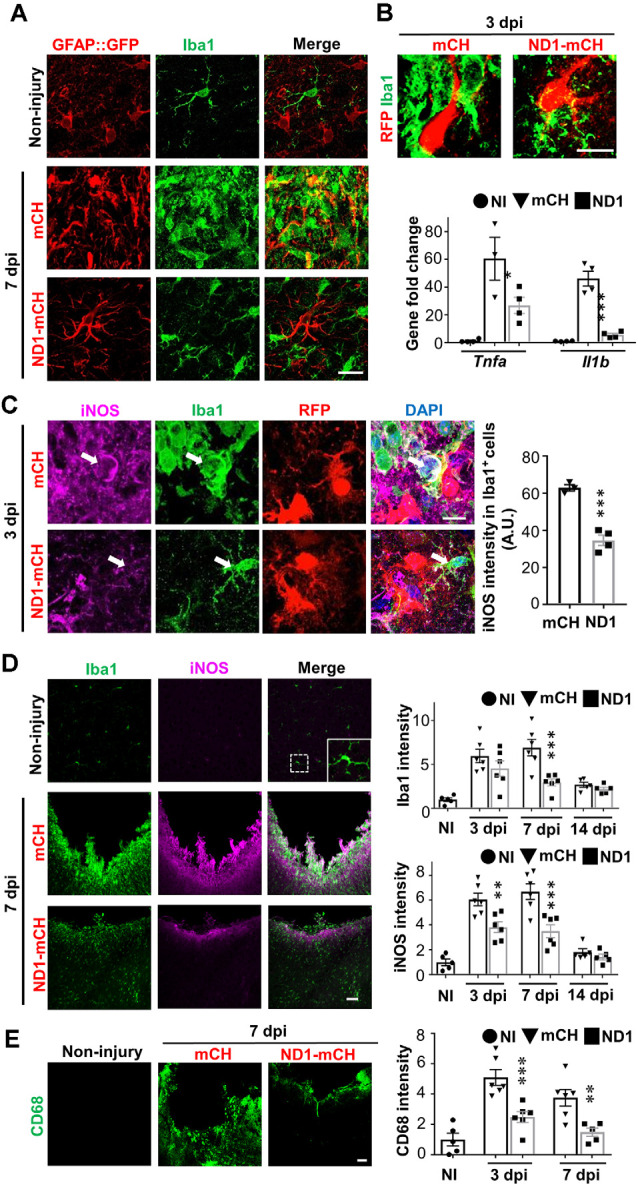
NeuroD1-treatment attenuates microglial inflammatory responses. **(A)** Microglia (Iba1, green) in non-injured brains displaying ramified branches (top row), but showing hypertrophic amoeboid shape in stab-injured areas (middle row, 7 dpi). In NeuroD1-infected injury areas, however, microglia returned to ramified morphology again (bottom row). Such morphological changes of microglia coincided with the morphological changes of astrocytes (GFAP::GFP labeling in the left column). Scale bar = 20 μm. **(B)** Representative images showing a close look of the microglia morphology (Iba1, green) contacting mCherry-infected astrocytes (left panel, red) or NeuroD1-mCherry infected astrocytes (right panel, red) at 3 dpi. Note that microglia showed clear morphological difference when contacting the NeuroD1-infected astrocytes as early as 3 dpi. Scale bar = 20 μm. Lower bar graph illustrating the gene expression level of inflammatory factors *Tnfa and Il1b* significantly increased in stab-injured cortices compared to non-injured cortical tissue, but such increase was greatly reduced in NeuroD1-infected injury areas (3 dpi). *n* = 4 mice. Each dot represents one animal. *P < 0.05, ***P < 0.001, one-way ANOVA followed with Sidak’s test. **(C)** Representative images illustrating many inflammatory M1 microglia labeled by nitric oxide synthase (iNOS) with amoeboid morphology in the control-AAV infected injury areas (upper panels, 3 dpi). In contrast, microglia in close contact with the NeuroD1-infected astrocytes showed much lower iNOS expression with ramified morphology (lower panels, arrow; 3 dpi). Scale bar = 10 μm. Quantitative analysis showing a significant reduction of the iNOS signal of Iba1^+^ cells in close contact with NeuroD1-infected cells. *n* = 3–4 mice per group. Each dot represents one animal. ***P < 0.001, Student’s *t*-test. **(D)** Representative images illustrating lack of iNOS signal in non-injured brains (upper panels), but high Iba1 and iNOS signal in stab-injured cortical tissue (middle panels, 7 dpi). However, in NeuroD1-infected cortices, both Iba1 and iNOS signals reduced significantly (bottom panels, 7 dpi). Scale bar = 50 μm. Right bar graphs, quantitative analyses showing the immunofluorescent signal of Iba1 and iNOS in non-injured (white bar), mCherry control (black bar), or NeuroD1 AAV-infected cortices (gray bar) at 3, 7, and 14 dpi. **P < 0.01, ***P < 0.001. Two-way ANOVA followed by Bonferroni *post hoc* tests. *n* = 5–6 mice per group. Each dot represents one animal. **(E)** Representative images showing the immunoreactivity of CD68, a macrophage marker, significantly reduced in NeuroD1-infected cortical tissues. Scale bar = 50 μm. Right bar graph showing a quantitative analysis result, which revealed a significant reduction of CD68 fluorescent signals in NeuroD1-infected cortices (gray bar) at 3 and 7 dpi. *n* = 5–6 mice per group. Each dot represents one animal. **P < 0.01, ***P < 0.001, two-way ANOVA plus Bonferroni *post hoc* test.

It is reported that toxic A1 astrocytes are activated by cytokines such as IL-1α, TNF, and C1q secreted by reactive microglia (Liddelow et al., 2017). Conversely, reactive astrocytes also secret cytokines such as TGFβ, CXCL10, CLC2, ATP, C3, ORM2 to modulate microglia (Chung and Benveniste, 1990; Norden et al., 2014). Here, we investigated what kind of impact would AtN conversion have on microglia. Compared to the resting microglia in non-injured brains (Figure 4A, top row), stab injury-induced reactive microglia that were hypertrophic and amoeboid-shape (Figure 4A, middle row). As expected, both microglia and astrocytes were highly proliferative after stab injury as shown by BrdU labeling (Supplementary Figures 5A–C), but no newborn neurons were detected in the adult mouse cortex after stab injury (Supplementary Figure 5D). In NeuroD1-infected areas, however, microglia morphology resembled closer to the resting microglia with ramified processes (Figure 4A, bottom row). Such morphological change started as early as 3 dpi, as shown in Figure 4B (top row), where microglia contacting NeuroD1-infected astrocytes showed more processes and less amoeboid shape compared to the microglia contacting mCherry-infected astrocytes. RT-PCR analysis revealed that the cytokines TNFα and IL-1β were both significantly increased after stab injury, but both attenuated in the NeuroD1 group (Figure 4B, bar graph, one-way ANOVA followed by Turkey’s test, *P < 0.05, ***P < 0.001, *n* = 4 pairs). Such a dramatic decrease of cytokines during AtN conversion may explain why microglia were less reactive in the NeuroD1 group. Consistently, toxic M1 microglia that were immunopositive for inducible nitric oxide synthase (iNOS) showed a significant reduction in the NeuroD1 group compared to the control group (Figure 4C, Student’s *t*-test, ***P < 0.001, *n* = 4 pairs). Note that the reduction of iNOS-labeled M1 microglia coincided with the reduction of toxic A1 astrocytes, as detected at 3 dpi after NeuroD1 infection, suggesting an intimate interaction between astrocytes and microglia (Volterra and Meldolesi, 2005). At 7 dpi, compared to the control group, the reduction of iNOS in the NeuroD1 group was even more significant, accompanied with a reduction of ionized calcium-binding adaptor molecule 1 (Iba1) signal as well (Figure 4D, two-way ANOVA, **P < 0.01, ***P < 0.001, *n* = 6 pairs at 3 dpi and 7 dpi, *n* = 5 pairs at 14 dpi). Besides, stab injury also induced a remarkable increase in the expression level of CD68, a marker for macrophages and monocytes as well as some reactive microglia (Figure 4E). However, in NeuroD1-infected areas, the expression level of CD68 was significantly attenuated (Figure 4E, bar graph, two-way ANOVA, **P < 0.01, ***P < 0.001, *n* = 6 pairs). Together, these results suggest that accompanying AtN conversion, toxic M1 microglia are reduced and neuroinflammation is alleviated.

### Astrocyte-Blood Vessel Interaction During *In vivo* Cell Conversion

**Figure 5:**
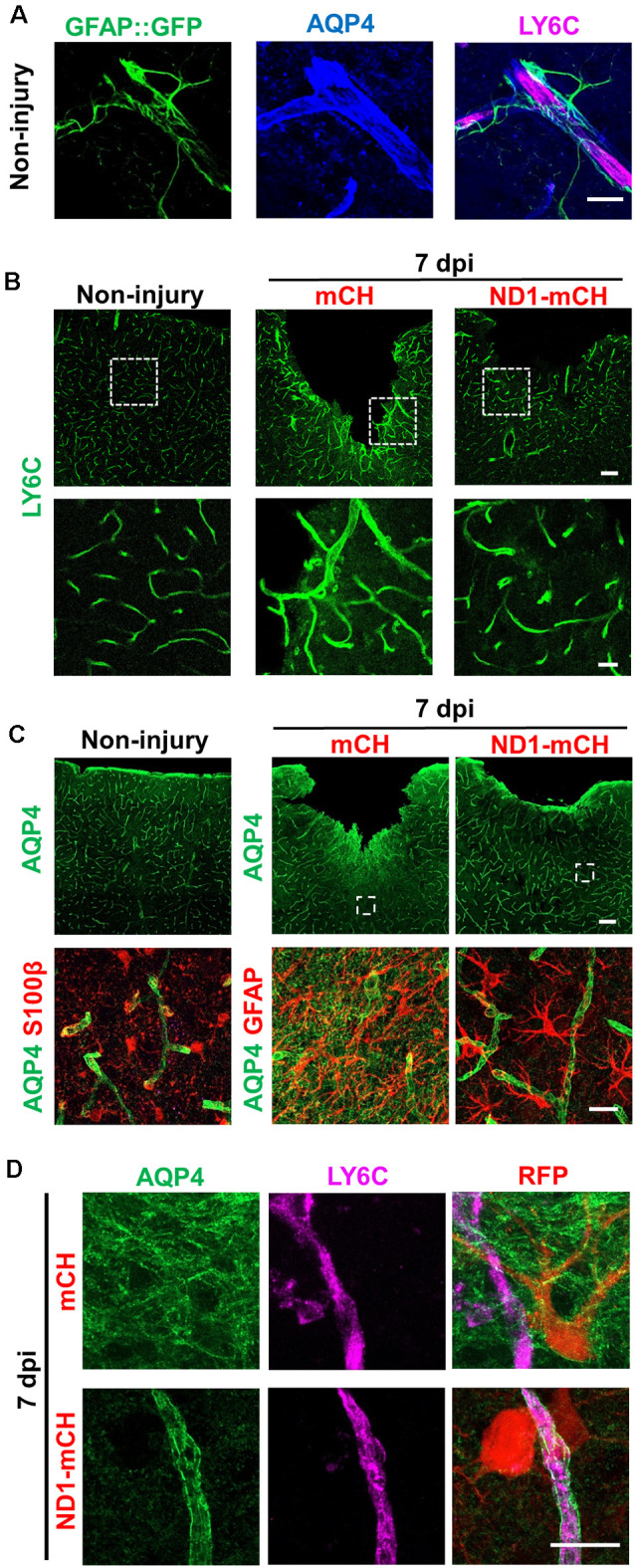
Repair of blood vessels and blood-brain-barrier (BBB) after stab injury through NeuroD1-mediated *in vivo* cell conversion. **(A)** Representative images showing the astrocyte-vascular unit in the non-injured mouse cortex. Astrocytes (green, labeled by GFAP::GFP) send their endfeet wrapping around blood vessels (magenta, labeled by LY6C, a vascular endothelial cell marker). Water channel protein aquaporin 4 (AQP4, was highly blue) concentrating at the astrocytic endfeet in resting state, which wrapped around the blood vessels. Scale bar = 20 μm. **(B)** Representative images in lower magnification (top row) and higher magnification (bottom row) showing the blood vessel morphology disrupted by stab injury. In NeuroD1-infected areas, the hypertrophic blood vessel morphology was partially reversed, closer to the ones in non-injured brains. Scale bars = 100 μm (low mag), 20 μm (high mag). **(C)** AQP4 signal in non-injured (Left column) and injured cortex (right two columns). Top row, low magnification images; Bottom row, high magnification images. Note that the AQP4 signal was typically concentrating at the endfeet of astrocytes wrapping around blood vessels (left column), but dissociated from the blood vessels after injury (middle column). Right column illustrating in NeuroD1-infected areas, the AQP4 signal (green) was re-associated with blood vessels. Scale bars = 100 μm (top row), 20 μm (bottom row). **(D)** Enlarged images to further illustrate the relationship between AQP4 and blood vessels (labeled by LY6C) in control AAV- vs. NeuroD1-treated injured areas. Top row, in control AAV mCherry-infected areas, AQP4 signal (green) was widely distributed in the injured areas and disassociated from blood vessels (magenta, LY6C). Bottom row, in NeuroD1-infected areas, the AQP4 signal (green) was re-associated with blood vessels (magenta, LY6C). Scale bar = 20 μm.

One important function of astrocytes in the brain is to interact with blood vessels and contribute to BBB to prevent bacterial and viral infection and reduce chemical toxicity (Obermeier et al., 2013). Breakdown of BBB will result in the leakage of a variety of biological and chemical agents into the parenchymal tissue, contributing to the secondary injury (Bush et al., 1999). In a healthy brain, BBB is tightened by astrocytic endfeet wrapping around the blood vessels (Figure 5A). Comparing to the evenly distributed blood vessels (labeled by endothelial marker Ly6C) in non-injured brains (Figure 5B, left panels), stab injury caused blood vessels to swollen (Figure 5B, middle panels). In the NeuroD1-treated group, however, blood vessels exhibited less hypertrophic morphology and closer to the ones in healthy brains (Figure 5B, right panels). Accompanying altered blood vessel morphology after stab injury, we also found a disruption of BBB, as evident by the mislocalization of the aquaporin-4 (AQP4) signal. AQP4 is a water channel protein, normally concentrating at the endfeet of astrocytes at resting state wrapping around blood vessels (see Figures 5A,C). After stab injury, the AQP4 signal dissociated from blood vessels and instead distributed throughout the injury areas (mCherry group in Figures 5C,D, top row). In NeuroD1-treated areas, the AQP4 signal showed re-association with blood vessels, returning to the normal state (Figure 5C, right column, and Figure 5D, bottom row). Note that the astrocytic morphology also looked much less reactive in the NeuroD1-treated group: the astrocytes in the mCherry-infected control group were hypertrophic and their processes were disorganized and often overlapped with each other; whereas the astrocytes in the NeuroD1 group showed well-branched processes and maintained their territories (Figure 5C, bottom right panel). Together, these results suggest that after NeuroD1-mediated cell conversion, astrocytes interact with blood vessels again to restore the broken BBB caused by the neural injury.

### Tissue Repair After NeuroD1-Mediated Neuronal Conversion

**Figure 6:**
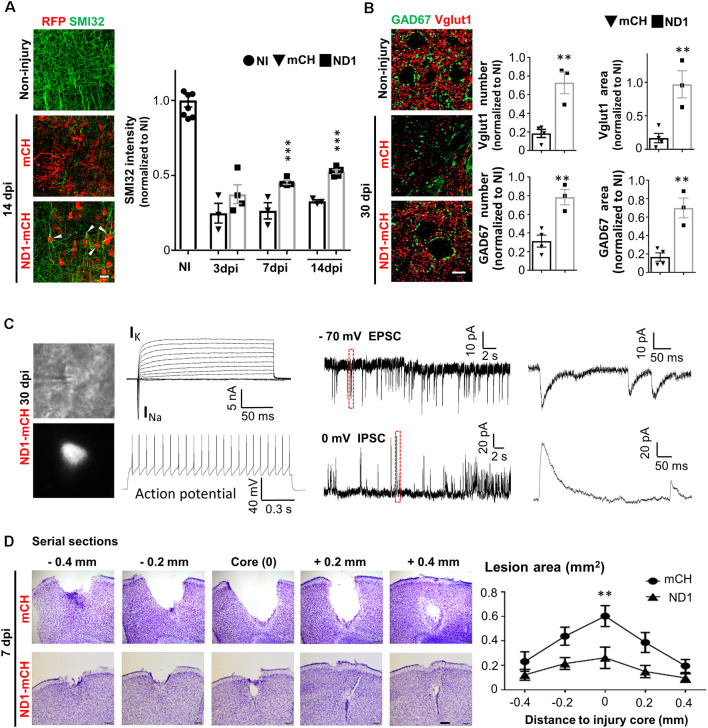
Tissue repair after NeuroD1-mediated AtN conversion. **(A)** Left images showing dendritic marker SMI32 (green) drastically reduced after stab injury (middle panel), but significantly rescued in NeuroD1-infected injury regions (bottom panel). Note that the NeuroD1-converted new neurons (red, bottom panel) were co-labeled by SMI32, indicating the newly generated neurons contributing to neural repair. Right bar graph illustrating the SMI32 signal partially recovered at 7 and 14 dpi. Scale bar = 20 μm. *n* = 3–4 mice per group. ***P < 0.001, two-way ANOVA plus Bonferroni *post hoc* test. **(B)** Rescue of synaptic loss after stab injury by NeuroD1-mediated cell conversion. Left images showing both glutamatergic synapses (red, Vglut1) and GABAergic synapses (green, GAD67) were significantly reduced after stab injury (middle panel, 30 dpi). However, after NeuroD1-treatment, both glutamatergic and GABAergic synapses showed significant recovery. Scale bar = 20 μm. Right bar graphs showing quantitative analysis results. Note that both the number of synaptic puncta and the covered area were rescued in NeuroD1-treated injury areas. *n* = 4–5 mice per group. ***P < 0.001, two-way ANOVA plus *post hoc* test. **(C)** Electrophysiological analysis demonstrating that the NeuroD1-converted neurons were functional. Left images showing patch-clamp recordings performed on NeuroD1-mCherry-infected cells in cortical slices (30 dpi). Right traces showing a typical recording of large Na^+^ and K^+^ currents, repetitive action potentials, and excitatory (EPSC) and inhibitory (IPSC) postsynaptic currents. *n* = 15 cells from three mice. **(D)** Rescue of tissue loss by NeuroD1-mediated AtN conversion. Top row illustrating cortical tissue damage induced by stab injury with Nissl staining in serial brain sections across the injury core (7 dpi) of one control mouse. Bottom row illustrating much less tissue loss in a NeuroD1-treated mouse. Scale bar = 200 μm. Right line graph showing the quantitative analysis result (*n* = 5 mice for both control and NeuroD1 groups). Across the entire injury areas, cortical tissue loss was significantly rescued throughout the NeuroD1-infected regions. The tissue areas absent of crystal violet signals were quantified. **P < 0.01, two-way ANOVA followed with Bonferroni *post hoc* test.

With a substantial change in the glial environment during and after NeuroD1-mediated AtN conversion, we further investigated neuronal properties such as dendritic morphology, synaptic density, and electrophysiological function in the injured areas. Stab injury resulted in severe dendritic damage as expected, shown by a significant decrease of dendritic marker SMI32 in injured areas (Figure 6A, left middle images, green signal). In NeuroD1-infected areas, however, we observed a significant increase in dendritic signal SMI32 (Figure 6A, bottom image). Quantitatively, neuronal dendrites labeled by SMI32 were severely injured after stab lesion, decreasing to 25% of the non-injured level, but NeuroD1 treatment rescued the dendritic signal to over 50% of the non-injured level at 14 dpi (Figure 6A; bar graph, two-way ANOVA, ***P < 0.001, *n* = 4 pairs). Many SMI32-labeled dendrites were colocalized with NeuroD1-mCherry signals (Figure 6A; arrowheads), indicating a significant contribution of the converted neurons to neural repair. Consistent with the dendritic damage, stab injury also caused a severe reduction of synapses in the injured areas as shown by glutamatergic synapse marker vesicular glutamate transporter 1 (vGluT1) and GABAergic synapse marker glutamic acid decarboxylase 67 (GAD67; Figure 6B, left images). After NeuroD1 treatment (30 dpi), both glutamatergic and GABAergic synaptic density in the injured areas showed a significant increase compared to the control group (Figure 6B, bar graphs).

Next, we investigated whether the NeuroD1-converted neurons are physiologically functional. Cortical brain slices were prepared and patch-clamp recordings were performed on the NeuroD1-mCherry converted neurons (Figure 6C, left images). After 1 month of conversion, the NeuroD1-mCherry positive neurons showed large sodium and potassium currents (13/15 recorded cells) and repetitive action potentials (7/10 cells; Figure 6C, left traces). We also recorded both glutamatergic synaptic events in 10/13 cells (frequency: 0.96 ± 0.5 Hz; amplitude: 24.4 ± 6.3 pA; holding potential = −70 mV) and GABAergic synaptic events in 9/13 cells (frequency: 0.74 ± 0.16 Hz; amplitude: 55.9 ± 7.7 pA; holding potential = 0 mV; Figure 6C, right traces), consistent with the recovery of vGluT1 and GAD67 immuno-puncta shown in Figure 6B.

We have repeatedly observed a significant tissue loss after severe stab injury during a variety of immunostaining analysis, but a remarkable tissue repair in the NeuroD1-treated group (Figures 2A,C, 3E, 4D,E, 5B–D). To quantitatively assess the level of tissue loss, we performed serial cortical sections with Nissl staining around the injury core in both the mCherry control group and the NeuroD1 treatment group. Nissl staining revealed a large tissue loss across the serial brain sections in the mCherry control group after stab injury (Figure 6D, top row). In contrast, the NeuroD1-treatment group showed much less tissue loss across all brain sections (Figure 6D, bottom row). Quantitatively, the tissue loss around the injury areas in the NeuroD1 group was significantly reduced by 60% compared to the control group (Figure 6D, right line graph, Two-way ANOVA, **P < 0.01, *n* = 5 pairs). Together, these results suggest that converting reactive astrocytes into functional neurons significantly preserves neural tissue.

### Converting Reactive Astrocytes Into Neurons After Glial Scar Formation

**Figure 7:**
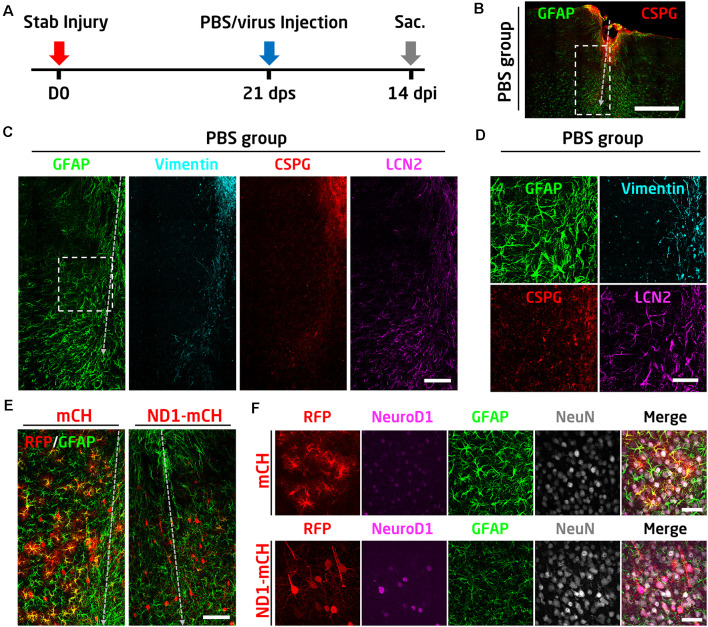
NeuroD1 can convert reactive astrocytes into neurons after glial scar formation. **(A)** Schematic illustration of the timeline of experimental design. The viral injection was carried out at 21 days after a stab lesion when the glial scar being well established. PBS was injected as a control to assess glial scar formation. Mice were sacrificed for data analysis at 14 days after viral injection. **(B)** Representative image illustrating severe tissue damage in the mouse cortex at 35 days after stab injury, with PBS, injected at 21 days following stab injury as a control. Scale bar: 500 μm. **(C)** Enlarged images from panel B (dashed rectangle) showing densely packed hypertrophic reactive astrocytes around the stab lesion area. GFAP and LCN2 were highly expressed in the reactive astrocytes. Vimentin and CSPG were also upregulated in the injured area. Scale bar: 100 μm. **(D)** High magnification images from panel C (dashed square) showing the upregulation of GFAP, vimentin, CSPG, and LCN2 after stab injury. Scale bar: 50 μm. **(E)** Comparison of the virally infected lesion areas between mCherry control (left) and NeuroD1-treated group. Scale bar: 100 μm. **(F)** High magnification images showing the virally infected cells in both control and NeuroD1 groups. In the control group, mCherry-expressing cells showed reactive astrocyte morphology with a high expression level of the GFAP signal. In contrast, NeuroD1-infected cells showed neuronal morphology, and the astrocytes in the NeuroD1 group were less hypertrophic with a low level of GFAP signal compared to the control group. Scale bar: 50 μm.

In the above experiments, we have demonstrated that early intervention after traumatic stab injury (at 4 days after stab lesion) can effectively convert reactive astrocytes into functional neurons and essentially limiting the glial scar formation. One remaining question is whether the reactive astrocytes in the well established glial scar tissue can still be converted into neurons. To answer this question, we injected AAV viruses at 21 days after stab lesion (21 dps; Figure 7A), when the glial scar is known to be formed at this late stage. As a control, PBS was injected at 21 dps to assess the glial scar formation following severe stab lesion (Figures 7A–D). There was a significant tissue loss after 1 month of stab injury (Figure 7B). Immunostaining results showed that the astrocytes in the lesion areas were highly reactive, showing hypertrophic morphology with an abnormally high level of GFAP and vimentin, as well as inflammatory factors such as CSPG and LCN2 (Figures 7C,D). The highly reactive astrocytes were similarly seen in the control mCherry virus-injected areas (Figures 7E,F, mCherry group, high GFAP signal). However, in NeuroD1-mCherry infected areas, astrocytes became much less reactive as shown by low GFAP signal and less hypertrophic morphology (Figure 7F). More importantly, the majority of NeuroD1-mCherry infected cells became NeuN-positive neurons (Figures 7E,F). Therefore, we conclude that the reactive astrocytes before and after glial scar formation can both be converted into neurons.

## Discussion

In this study, we demonstrate that NeuroD1-mediated astrocyte-to-neuron (AtN) conversion not only regenerates functional neurons but also improves glial landscape and restore the neuron:astrocyte ratio in the injury sites. Importantly, this study has answered three critical questions regarding the *in vivo* AtN conversion technology: (1) astrocytes are not depleted after conversion; (2) glial scar tissue can be reversed back to neural tissue to achieve real tissue repair; and (3) altered neuron-glia ratio after an injury can be rebalanced after conversion. Before converting into neurons, ectopic expression of NeuroD1 in reactive astrocytes can reduce the expression level of toxic A1 marker genes and hence reduce the released toxic cytokines. Meanwhile, toxic M1 microglia and neuroinflammation in the injury area are attenuated, and blood vessels and BBB are restored after NeuroD1-mediated AtN conversion. Together, AtN conversion results in more functional neurons, less reactive glial cells, better blood vessels, and rebalanced neuron:astrocyte ratio in the injury area, leading to a reversal of glial scar tissue back to neural tissue which is a fundamental path toward neural tissue repair.

### Rebalancing Neuron to Glia Ratio After Injury

Glial cells, in particular astrocytes, closely interact with neurons to provide structural, metabolic, and functional support (Nedergaard et al., 2003). A delicate balance between neurons and glia ensures normal brain function. Injury to brain tissue leads to irreversible neuronal loss accompanied by glial proliferation. As a result, the delicate balance between neurons and glia is altered and brain functions impaired. Although the functional role of astrocytes in neural injury has been extensively examined in previous studies, rebalance of the neuron:glia ratio after an injury has not been achieved successfully before. This may be partly because previous technologies are not capable of simultaneously regenerating new neurons and reducing reactive glial cells in the injury sites. For example, transplantation of exogenous neuroprogenitor cells can generate new neurons but cannot reduce reactive glial cells (Toft et al., 2007; Lu et al., 2012; Péron et al., 2017). On the other hand, ablation of astrocytes can reduce the number of glial cells but cannot generate new neurons (Anderson et al., 2016). Other approaches attempting to attenuate glial scar such as using chondroitinase ABC to reduce CSPG level cannot generate new neurons (Sekiya et al., 2015). Compared to these previous approaches targeting either neurons or glial cells in a separate manner, our *in vivo* AtN conversion technology is a “one stone-two birds” approach, not only generating new neurons but also reducing reactive glial cells at the same time. Hence, we can quickly rescue the neuron to astrocyte ratio after brain injury to a level much closer to the resting state after AtN conversion. Accompanying such a remarkable increase of neuron:astrocyte ratio in the injury sites, neuronal dendrites, and synaptic density also increase significantly after AtN conversion. Therefore, restoration of the neuron:glia ratio after an injury is critical for brain repair.

### Astrocytes Not Depleted but Rather Repopulated After Neuronal Conversion

As the most abundant cell type in the mammalian CNS, astrocytes influence brain functions by providing trophic support to neurons, regulating BBB, modulating immune cells, and controlling synapse formation. It is well established that after injury or disease, reactive astrocytes undergo dramatic morphological change and express a high level of GFAP and cytokines such as TNFα and Lcn2 in the CNS (Zamanian et al., 2012). Reactive astrocytes and microglia can release a high amount of glutamate and ATP to induce neurotoxicity (Takeuchi et al., 2006; Orellana et al., 2011). However, the precise function of reactive astrocytes after an injury is still under debate due to their double-faceted roles. On one hand, the inhibitory effect of reactive astrocytes on axon regeneration has been widely reported (Silver and Miller, 2004; Yiu and He, 2006; Cregg et al., 2014). Reactive astrocytes also produce pro-inflammatory cytokines that exacerbate CNS injury (Brambilla et al., 2005; Pineau et al., 2010; Sofroniew, 2015). On the other hand, ablation of scar-forming astrocytes after an injury has been reported to impair axon regeneration and worsening the injury areas (Bush et al., 1999; Anderson et al., 2016). It is important to point out that these results are based on the ablation of scar-forming astrocytes, which causes massive astrocytic death in the injury areas and will inevitably trigger massive neuroinflammation from reactive microglia. This is indeed what has been observed when astrocytes are killed (Bush et al., 1999; Anderson et al., 2016). Over-activation of microglia and macrophages can induce profound neurotoxicity and axonal retraction through both physical interaction and inflammatory responses (Horn et al., 2008; Hu et al., 2015; Silver, 2016). Besides, astrocytes provide a variety of support to normal neuronal functions, ranging from neurotrophic nourishing to neurotoxin removal as well as regulating synaptic structure and functions (Allen and Barres, 2009). Therefore, it is not surprising that killing astrocytes will further deteriorate the microenvironment of injury areas if neurons are deprived of astrocytic support. In contrast to the killing astrocyte approach, our *in vivo* AtN conversion approach not only regenerates new neurons but also regenerates new astrocytes, as shown by increased proliferation following conversion. Thus, astrocytes are not depleted by conversion but rather repopulated in the injured areas after AtN conversion.

Why killing astrocytes results in worsening of neural injury, whereas converting astrocytes into neurons repairs damaged neural tissue? The fundamental difference is the presence of new neurons and new astrocytes in the injured areas following AtN conversion. When transgenic mice were used to impair normal astrocyte functions, inflammation was increased in the injury areas and neurons could not get the necessary support from the impaired astrocytes (Anderson et al., 2016). In contrast, when reactive astrocytes are converted into neurons, the remaining astrocytes can proliferate and repopulate themselves in the injury areas. Therefore, AtN conversion creates an entire new microenvironment in the injury sites, which includes new neurons, new astrocytes, new blood vessels, together with reduced microglia and reduced inflammatory cytokines. It is the summation of all these effects together, rather than any single factor among them, that makes ours *in situ* AtN conversion approach very powerful for brain repair. It is also worth mentioning that NeuroD1 not only can convert reactive astrocytes into neurons shortly after the injury occurred (4 dps) but also can convert the scar-forming astrocytes long after the injury (21 dps) into neurons, suggesting that such *in vivo* conversion approach can be potentially applied with a broad time window for therapeutic interventions. Interestingly, different from reactive astrocytes, non-reactive astrocytes can also be converted into neurons but at lower efficiency, suggesting that resting astrocytes might be more resistant to conversion (Brulet et al., 2017).

One of the new insights gained from this study is that in addition to neural injury that can stimulate astrocytic proliferation, we now demonstrate that AtN conversion in the adult brain can also trigger astrocytic proliferation. It is known that adult astrocytes in the resting state do not proliferate unless under the condition of injury or diseases. Why astrocytes proliferate after AtN conversion? We hypothesize that such proliferation may be due to the loss of direct contact between astrocytes, but the precise underlying mechanism is an interesting topic to be further explored in future studies. It may be worth mentioning that a previous report found that mild stab injury in the brain resulted in less astrocytic proliferation (Bardehle et al., 2013). In the current study, we purposely used a large needle (outer diameter 0.95 mm) to induce severe stab injury, resulting in a clear tissue loss in the mouse motor cortex. Accordingly, we found a significant number of proliferating astrocytes in the injured areas. Therefore, different levels of neural injury or different disease models may result in different levels of astrocytic proliferation.

The stab injury in this study does not induce obvious behavioral deficits. Therefore, no behavioral tests were performed to evaluate functional rescue. The focus of this study is to investigate the rescue of the neuron to astrocyte ratio after AtN conversion, which provides a fundamental cellular basis for tissue repair.

### Attenuation of Neuroinflammation and Repair of BBB

It is unexpected when we detect a drastic decrease of microglia and neuroinflammatory factors in the injury areas following NeuroD1-mediated AtN conversion. If astrocytic and neuronal changes are directly associated with AtN conversion, the change in microglia is an indication of a non-cell-autonomous impact on the local environment. Microglia act as neuroprotective cells and play a pivotal role in immune defense in the brain by constantly surveying brain tissue to identify any potential damage for immediate repair. On the other hand, excessive activation of microglia often leads to secondary neuronal damage due to their secretion of neurotoxic cytokines, inflammatory factors, and reactive oxygen spices (Neher et al., 2013; Hu et al., 2015). Therefore, like reactive astrocytes, reactive microglia also act as a double-edged sword, with both positive and negative effects on injured nerve tissue. How to control the microglia at an optimal level is pivotal for neural repair. One major challenge is to find out what is the best time window to stop microglial over-reactive response during the repair. In the current study, we were surprised to observe diminished microglial reactivity and a drastic reduction of inflammatory factors such as TNFα and interleukin 1β (IL-1β) accompanying AtN conversion. We hypothesize that such microglial change may be related to the reduction of reactive astrocytes because astrocytes can modulate microglial activity through a variety of factors including ATP, Ca^2+^, complement (C3), chemokines, and plasma protein ORM2 (Schipke et al., 2002; Davalos et al., 2005; Zamanian et al., 2012; Clarner et al., 2015; Lian et al., 2016; Jo et al., 2017). Therefore, by converting reactive astrocytes into neurons, NeuroD1 treatment indirectly regulates the reactivity of microglia and reduces neuroinflammation.

Another indirect impact of AtN conversion is the BBB repair in NeuroD1-infected areas. Astrocytes actively participate in the maintenance of BBB integrity by physically contacting blood vessels with endfeet and secreting growth factors such as vascular endothelial growth factor A (VEGFA), basic fibroblast growth factor 2 (FGF2), interleukin 6 (IL-6), and glia-derived neurotrophic factor (GDNF) to promote angiogenesis (Obermeier et al., 2013). Brain injury is associated with BBB breakdown (Shlosberg et al., 2010) because reactive astrocytes cannot maintain normal BBB structure and function in an injured environment (Fukuda and Badaut, 2012). This is confirmed in our study, as shown by the mislocalization of the AQP4 signal after stab injury, which indicates the dissociation of astrocytic endfeet from the blood vessels. What is unexpected is the reversal of the AQP4 signal back to the blood vessels after AtN conversion, suggesting that the astrocytic endfeet is re-associated with the blood vessels. The repair of BBB could also be a result of alleviation of inflammation, as microglial activation has been reported to impair BBB function by releasing cytokines (e.g., TNFα, IL1ß, and IFNγ; da Fonseca et al., 2014). While much more mechanistic studies are needed to interpret these discoveries, one critical insight gained from our studies is that AtN conversion is having a broad impact on the local brain environment beyond new neurons generated.

### Implications for Future Clinical Translations

One important issue regarding future clinical applications is that our AAV Cre-FLEX system is designed for long-term tracing the converted neurons for basic research purposes, and the Cre recombinase will not be applied to patients during clinical therapies where there is no need to trace the converted neurons. Another issue to be considered is the potential pre-existing AAV capsid-specific neutralizing antibodies in patients that may complicate the clinical design for each individual. For this particular reason, we believe that intracranial injection of AAV into the injured brain area to repair the local damaged tissue might be a better approach than the systemic injection through intravenous (i.v.) delivery route, at least at this moment before new serotype AAV emerges. Because local intracranial injection of AAV will use very low doses to reach a relatively high concentration in the local brain tissue; whereas i.v. injection of AAV will use a much higher dose in the circulation to reach a reasonable concentration in the brain that may trigger severe inflammation in the liver, spleen, kidney, etc. We have already performed non-human primate experiments where intracranial injection of AAV resulted in a nice viral infection in every monkey injected (Ge et al., 2019), suggesting that pre-existing AAV antibodies, if any, in the monkey brain may not be sufficient to antagonize the AAV injected locally.

This cell conversion technology is not intended for acute treatment within hours of neurotrauma but rather intended for neural regeneration and repair after days and weeks following neurotrauma. Therefore, our approach is complementary to the existing acute treatments, which are mainly focusing on neuroprotection and aiming to open a new avenue for long-term functional improvement through *in vivo* neuroregeneration approach. The current study provides a new angle to think about how to repair a damaged brain even after glia scar formation.

## Conclusions

Previous studies have well documented both positive and negative effects of glial scar formation on neural function recovery. On one hand, killing reactive astrocytes in the injury sites exacerbates the injury due to the loss of positive effect of astrocytes; on the other hand, the persistence of glial scar in the brain also inhibits functional recovery due to inhibition of neuronal regrowth. So far, previous studies have not been able to effectively remove glial scar inside the brain without causing additional impairment. Our *in vivo* cell conversion technology opens a new avenue to tackle glial scar from within the scar tissue without surgical resection or external cell transplantation. By directly converting reactive astrocytes into neurons in the injury sites *in situ*, our AtN conversion technology offers an effective way to reverse glial scar back to neural tissue without introducing any exogenous cells. Following the reduction of reactive astrocytes and regeneration of new neurons after AtN conversion, we detect a series of beneficial effects such as rebalancing neuron to astrocyte ratio, reduction of microglia, and neuroinflammation, increase of neuronal dendrites and synaptic density, and restoration of BBB. Therefore, *in vivo* cell conversion technology may provide a potential alternative approach for neural repair through reversing glial scar back to neural tissue.

## Data Availability Statement

All datasets presented in this study are included in the article/Supplementary Material.

## Ethics Statement

The animal study was reviewed and approved by the Pennsylvania State University IACUC.

## Author Contributions

GC supervised the entire project and wrote the manuscript together with LZ. LZ performed the majority of experiments and assembled most of the figures. ZL helped with experiments and data analysis, as well as making figures. ZG initiated earlier studies. ZP provided all the viruses. YB helped with mouse work. YC, FZ, AC, GM, GL, VS, and FW participated in some experiments and data analysis. All authors contributed to the article and approved the submitted version.

## Conflict of Interest

GC is a co-founder of NeuExcell Therapeutics Inc. The remaining authors declare that the research was conducted in the absence of any commercial or financial relationships that could be construed as a potential conflict of interest.

## Abbreviations

NeuroD1: neurogenic differentiation 1
AAV: adeno-associated virus
AtN: astrocyte-to-neuron
Cre: Cre recombinase
FLEX: flip-excision
GFAP: glial fibrillary acidic protein
NeuN: neuronal nuclei
CSPGs: chondroitin sulfate proteoglycans
LCN2: lipocalin-2
Iba1: Ionized calcium-binding adaptor molecule 1
iNOS: inducible nitric oxide synthase
IL-1β: interleukin-1β
BBB: blood-brain-barrier
BrdU: bromodeoxyuridine
dps: days post stab injury
dpi: days post-viral injection
AQP4: aquaporin-4
vGluT1: vesicular glutamate transporter 1
GAD67: glutamic acid decarboxylase 67 (GAD67).
