# Supplementary material for: Development of Neuroregenerative Gene Therapy to Reverse Glial Scar Tissue Back to Neuron-Enriched Tissue
Source: Front Cell Neurosci. 2020 Nov 5;14:594170. doi: 10.3389/fncel.2020.594170 (PMC7674596; doi:10.3389/fncel.2020.594170)
Supplement: Supplementary file 1 [file Data_Sheet_1.PDF]

Supplementary Material:

Development of Neuroregenerative Gene Therapy to Reverse Glial Scar Back to Neural Tissue

Lei Zhang et al., … Gong Chen

Supplementary Figures and Legends:

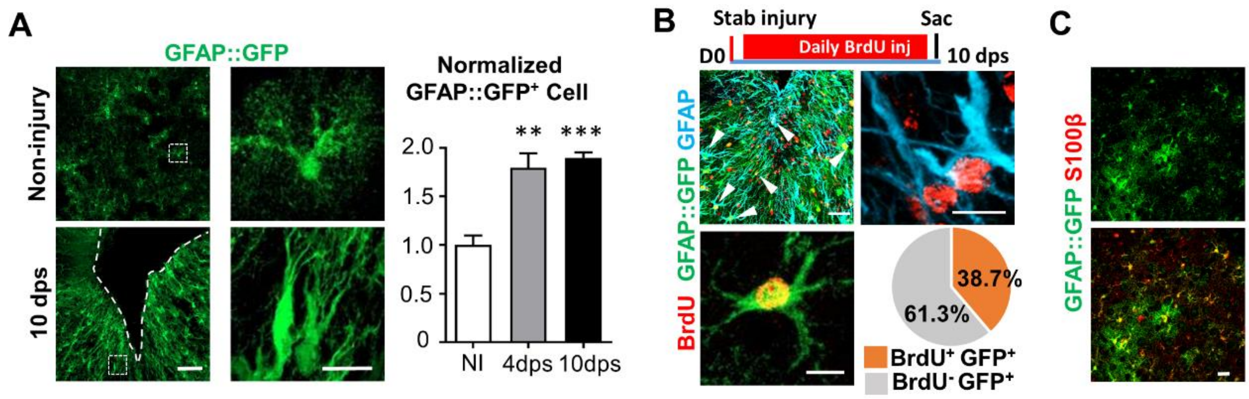

Figure S1. Significant proliferation of astrocytes after severe stab injury.

A. Severe stab injury in mouse motor cortex induced reactive astrocytes and tissue loss. Upper row illustrating normal astrocytes in non-injured cortical tissue of GFAP::GFP mice with elaborate processes and non-overlapping with their neighboring astrocytes. Bottom row showing a significant tissue loss induced by stab injury in the mouse motor cortex, and a large number of hypertrophic reactive astrocytes at 4 or 10 days post stab injury (dps). Scale bars = 100 µm (left low mag. panels), 20 µm (right high mag. panels). Right bar graph, quantitative analysis showing an increase of astrocytic number after stab injury. NI, non-injury. n = 4 mice. *** $P < 0.001$, Student's *t*-test.

B. Proliferation of astrocytes after stab injury. 5-Bromo-2′-deoxyuridine (BrdU) was injected intraperitoneally into GFAP::GFP mice daily until 10 dps. Many GFP^+ cells were co-labeled with BrdU (red) and GFAP (cyan), indicating cell division of astrocytes after stab injury. Quantitative analysis found that 38.7 ± 2.5 % of GFP^+ astrocytes were $BrdU^+$, suggesting a high proliferation

rate in the injury sites. n = 4 mice. Scale bars = 50 µm (top left), and 10 µm (top right and bottom left).

C. The GFP-positive cells in GFAP::GFP mice were confirmed to be astrocytes, as shown with another astrocyte marker S100β.

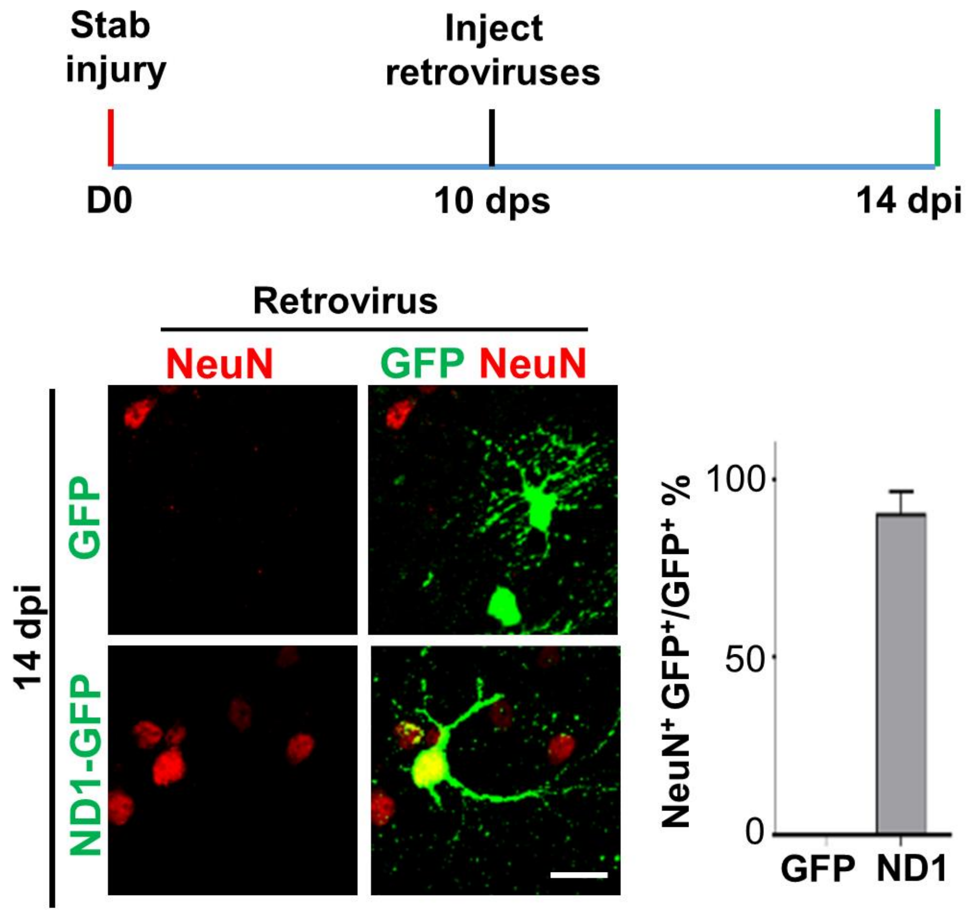

Figure S2. Overexpression of NeuroD1 by retroviruses efficiently converted reactive astrocytes into neurons after stab injury.

Retroviruses carrying CAG::NeuroD1-IRES-GFP or CAG::GFP (control) were injected into stab-injured motor cortex at 4 dps. At 14 days post viral injection (dpi), mice were sacrificed and subjected to immunostaining. The GFP-infected cells showed glial morphology and immunonegative for NeuN (top row), whereas the majority of NeuroD1-infected cells were $NeuN^+$ neurons (bottom row). Right bar graph showing ~90% of NeuroD1-infected cells were converted into neurons. Scale bar = 10 μm, n= 4 mice.

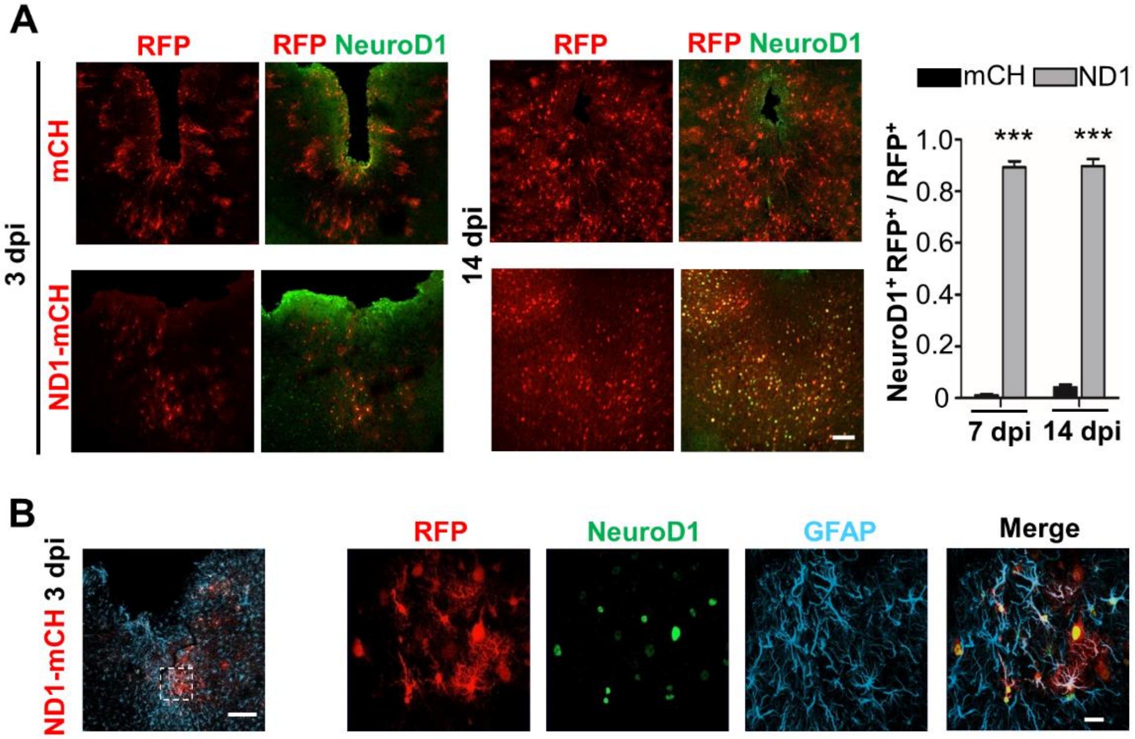

Fig. S3. Highly efficient and early expression of NeuroD1 in stab-injured areas using our AAV9 Cre-FLEX system.

A. Representative images (left panels) showing widespread AAV infection in the stab-injured cortical areas. Right bar graph, quantitative analysis showing ~90% of NeuroD1-mCherry infected cells expressed high level of NeuroD1. Scale bar = 100 μm. n = 4-6 mice per group. *** P < 0.001, one-way ANOVA plus Sidak's test.

B. Representative images showing early expression of NeuroD1 in infected astrocytes (3 dpi). Quantitatively, among NeuroD1-mCherry infected cells, 92.8 ± 2.8% are GFAP-positive astrocytes (cyan), and 87.4 ± 2.5% are positive for NeuroD1 (green), n = 6 mice.

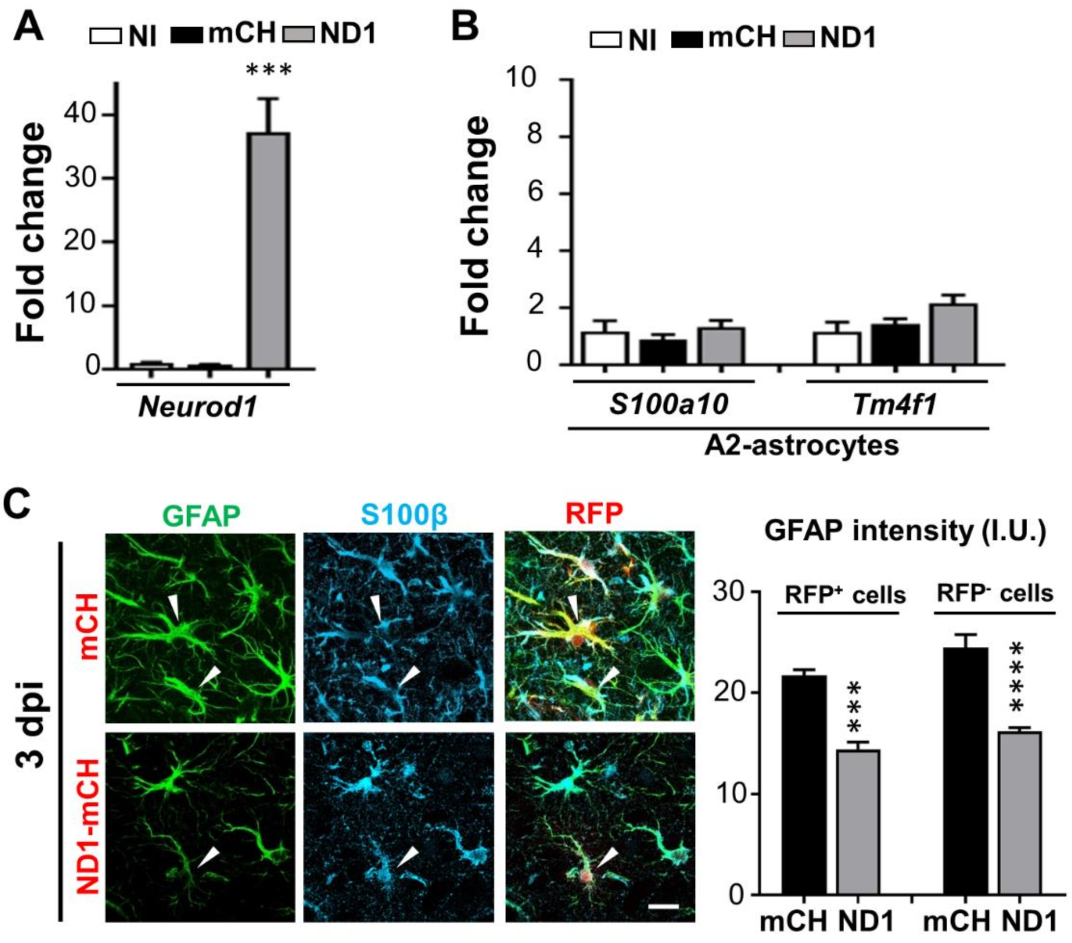

Fig. S4. Early effect of NeuroD1 in reducing GFAP expression after infecting astrocytes.

A. Quantitative real-time PCR (qRT-PCR) confirmed a drastic increase of NeuroD1 expression at 3 days post NeuroD1-AAV infection (3 dpi). n = 4 mice. *** $P < 0.001$, one-way ANOVA plus Sidak's test.

B. Quantitative RT-PCR results suggest no significant changes in the expression of A2-astrocytic markers *S100a10* or *Tm4f1*. n = 4 mice. No statistical significance. One-way ANOVA plus Sidak's test.

C. Left images illustrating less reactive morphology and less GFAP expression in NeuroD1-infected astrocytes (bottom row, arrowhead) and NeuroD1-non-infected astrocytes compared to those astrocytes in mCherry-infected brains (top row, arrowhead). Right bar graph showing quantitative results.

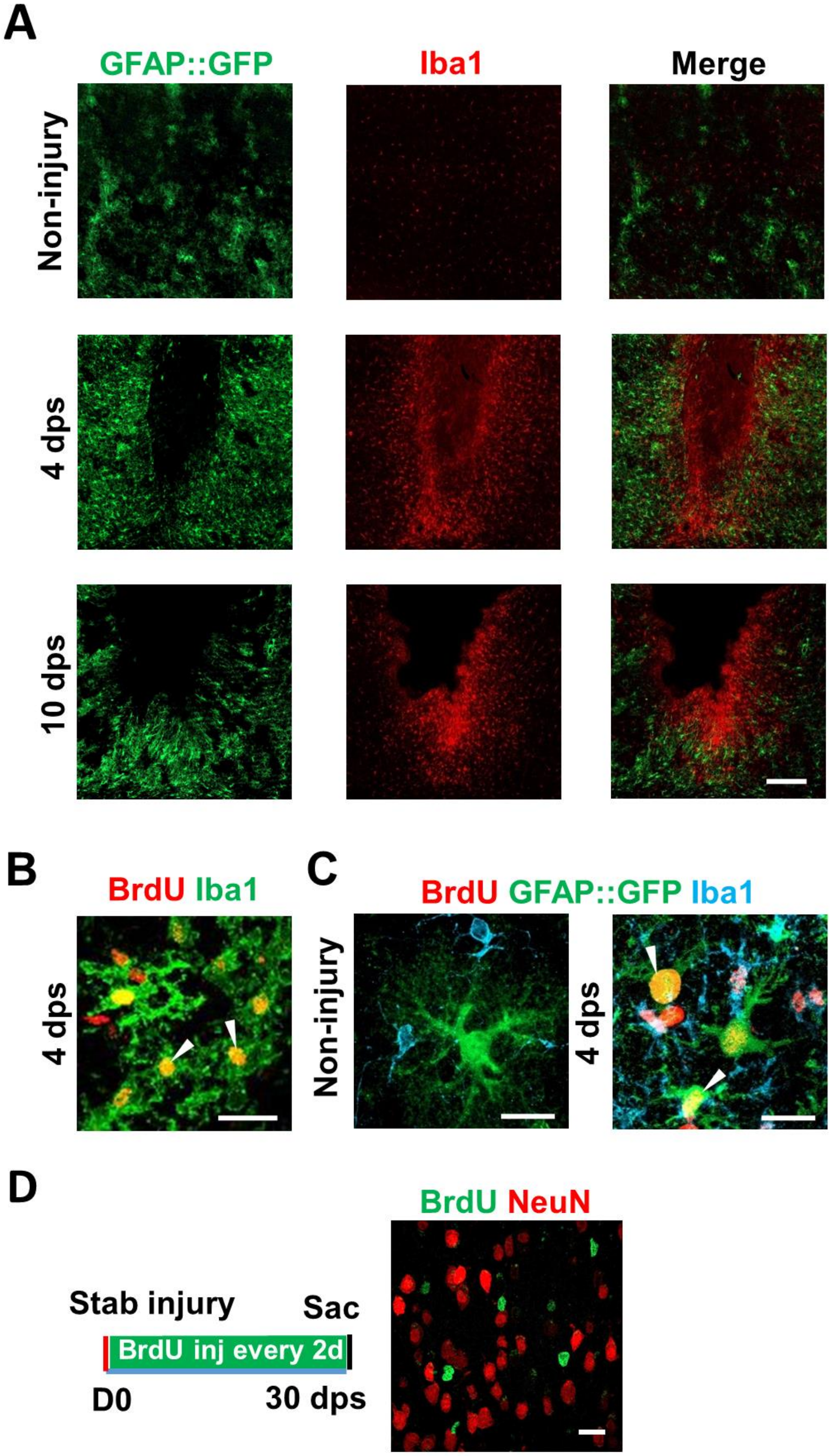

Fig. S5. Activation of microglia and astrocytes after stab injury but lack adult neurogenesis in the mouse cortex.

A. Representative images showing the drastic accumulation of microglia (Iba1, red) around the injured cortical areas at 4 and 10 days post stab injury. Note that astrocytes (GFP, green) were also activated around the injury site. Scale bar = 200 µm.

B. Proliferation of both microglia and astrocytes in the stab-injured mouse cortex. BrdU was applied daily after stab injury. Representative images showing BrdU (red) labeling in many Iba1$^+$ (green) cells, suggesting high proliferation of microglia after injury. Scale bar = 20 µm.

C. Left image showing resting microglia (cyan) and astrocytes (green) in non-injured GFAP::GFP mouse cortex. Right image illustrating after stab injury (4 dps), both astrocytes (green) and microglia (Iba1, cyan) were BrdU$^+$.

D. Very low internal neuroregeneration capability in the adult mouse cortex after stab injury. BrdU was applied every 2 days for 1 month to label the internal newborn neurons after stab injury. BrdU$^+$ cells are rarely co-labeled with NeuN (<1%), indicating very low endogenous adult neurogenesis in the mouse cortex. n = 3 mice. Scale bar = 20 µm.
